# Molecular Inferences Suggest Multiple Host Shifts of Rabies Viruses from Bats to Mesocarnivores in Arizona during 2001–2009

**DOI:** 10.1371/journal.ppat.1002786

**Published:** 2012-06-21

**Authors:** Ivan V. Kuzmin, Mang Shi, Lillian A. Orciari, Pamela A. Yager, Andres Velasco-Villa, Natalia A. Kuzmina, Daniel G. Streicker, David L. Bergman, Charles E. Rupprecht

**Affiliations:** 1 Centers for Disease Control and Prevention, Atlanta, Georgia, United States of America; 2 The Pennsylvania State University, State College, Pennsylvania, United States of America; 3 University of Georgia, Athens, Georgia, United States of America; 4 USDA/APHIS, Wildlife Services, Phoenix, Arizona, United States of America; Thomas Jefferson University, United States of America

## Abstract

In nature, rabies virus (RABV; genus *Lyssavirus*, family *Rhabdoviridae*) represents an assemblage of phylogenetic lineages, associated with specific mammalian host species. Although it is generally accepted that RABV evolved originally in bats and further shifted to carnivores, mechanisms of such host shifts are poorly understood, and examples are rarely present in surveillance data. Outbreaks in carnivores caused by a RABV variant, associated with big brown bats, occurred repeatedly during 2001–2009 in the Flagstaff area of Arizona. After each outbreak, extensive control campaigns were undertaken, with no reports of further rabies cases in carnivores for the next several years. However, questions remained whether all outbreaks were caused by a single introduction and further perpetuation of bat RABV in carnivore populations, or each outbreak was caused by an independent introduction of a bat virus. Another question of concern was related to adaptive changes in the RABV genome associated with host shifts. To address these questions, we sequenced and analyzed 66 complete and 20 nearly complete RABV genomes, including those from the Flagstaff area and other similar outbreaks in carnivores, caused by bat RABVs, and representatives of the major RABV lineages circulating in North America and worldwide. Phylogenetic analysis demonstrated that each Flagstaff outbreak was caused by an independent introduction of bat RABV into populations of carnivores. Positive selection analysis confirmed the absence of post-shift changes in RABV genes. In contrast, convergent evolution analysis demonstrated several amino acids in the N, P, G and L proteins, which might be significant for pre-adaptation of bat viruses to cause effective infection in carnivores. The substitution S/T_242_ in the viral glycoprotein is of particular merit, as a similar substitution was suggested for pathogenicity of Nishigahara RABV strain. Roles of the amino acid changes, detected in our study, require additional investigations, using reverse genetics and other approaches.

## Author Summary

Host shifts of the rabies virus (RABV) from bats to carnivores are important for our understanding of viral evolution and emergence, and have significant public health implications, particularly for the areas where “terrestrial” rabies has been eliminated. In this study we addressed several rabies outbreaks in carnivores that occurred in the Flagstaff area of Arizona during 2001–2009, and caused by the RABV variant associated with big brown bats (*Eptesicus fuscus*). Based on phylogenetic analysis we demonstrated that each outbreak resulted from a separate introduction of bat RABV into populations of carnivores. No post-shift changes in viral genomes were detected under the positive selection analysis. Trying to answer the question why certain bat RABV variants are capable for host shifts to carnivores and other variants are not, we developed a convergent evolution analysis, and implemented it for multiple RABV lineages circulating worldwide. This analysis identified several amino acids in RABV proteins which may facilitate host shifts from bats to carnivores. Precise roles of these amino acids require additional investigations, using reverse genetics and animal experimentation. In general, our approach and the results obtained can be used for prediction of host shifts and emergence of other zoonotic pathogens.

## Introduction

Rabies virus (RABV; genus *Lyssavirus*, Family *Rhabdoviridae*) circulates worldwide (except Australia, Antarctica and several insular territories) in a variety of carnivores, and in New World bats [1]. Circulation of RABV in Old World bats was suggested several times but not corroborated [2]. Bats are principal reservoir hosts of all lyssaviruses except Mokola virus (for which the principal hosts remain unknown). Generally, lyssaviruses are believed to have evolved originally in bats and later switched to carnivores [3]. Two such historical switches were inferred from viral phylogeny: one between lyssavirus genotypes (or species, following the present virus taxonomy), and the other within the rabies virus [4]. The latter suggestion was inferred from the observation that the raccoon RABV lineage is related phylogenetically to bat RABV lineages. As shown later, the south-central skunk RABV variant is monophyletic with the raccoon and bat RABV variants as well [5]. However, the major questions regarding these two switches remain unanswered: where, from which bat species, and into which terrestrial mammalian species the first switch occurred, given that bat-associated RABV has only been found in the New World, and non-RABV lyssaviruses only in the Old World, always in bats (except Mokola virus) [2], [3]. Moreover, there is no evidence that the raccoon or skunk RABV lineages facilitated transition of the virus to other carnivores, and the origins of viruses that represent multiple lineages of dog RABVs remain unknown.

The RABV genome consists of five genes, coding for structural proteins including N (nucleoprotein), P (phosphoprotein), M (matrix protein), G (glycoprotein), and L (large protein, or RNA-dependent RNA-polymerase). Each viral protein is multifunctional and significant for pathogenicity. The G protein is particularly important from the adaptive perspective, because it is responsible for recognition of host cell receptors and membrane fusion, promotes virus dissemination between infected cells, and stimulates host immune responses [6], [7]. The major forces of RABV evolution include point mutations, introduced by the viral polymerase during genome replication due to the lack of proofreading mechanisms, and purifying selection [8], [9]. The RABV populations demonstrate geographical variability, dominated by spatio-temporal separation (most notable in the widely distributed dog RABV lineages [10]), and compartmentalization of the diversity based on the particular reservoir species [11], [12].

Host adaptation mechanisms and host shifts/switches of lyssaviruses have not been explained. There was a suggestion that successful spill-over infections and host shifts within bats significantly depend on the host phylogeny (i.e., such events happen more frequently in evolutionary-related host species [12]). In addition, spill-over infections of bat RABVs in carnivores have been reported in the US often [13], [14], but these usually were single fatal events without perpetuation of transmission. Rare exceptions included a limited rabies outbreak in red foxes (*Vulpes vulpes*) on Prince Edward Island (Canada), previously free of carnivore rabies. Monoclonal antibody typing of the viruses, isolated from rabid foxes, suggested their likely origin from mouse-eared bats, *Myotis lucifugus* or *M. septentrionalis* [15]. More recently, a limited local outbreak, caused by a RABV variant associated with *T. brasiliensis* bats was documented in white-nosed coatis (*Nasua narica*) in Mexico [16].

Our study is dedicated to the series of rabies outbreaks in mesocarnivores caused by bat rabies viruses in the Flagstaff area (Coconino County) of northern Arizona, USA. The series was believed to have begun during 2001: 19 rabid striped skunks (*Mephitis mephitis*) were encountered during January–July. They all were infected with a RABV variant associated with big brown bats (*Eptesicus fuscus*) [17]. Before 2001, rabies in carnivores was documented only in the south of Arizona, where skunks and gray foxes (*Urocyon cinereoargenteus*) maintain circulation of their own specific RABV variants (the south-central skunk and the Arizona gray fox variants, respectively), but not in the northern Arizona. The Flagstaff outbreak triggered significant public health attention. Control measures included prohibiting relocation of nuisance skunks by pest control companies, comprehensive public education, pet rabies vaccine clinics, and a 90-day emergency quarantine requiring pets to be leashed or confined, and vaccinated. Additionally, 217 urban skunks were vaccinated parenterally with inactivated rabies vaccine during a 6-month phased program of trap, vaccinate, and release (TVR). The epizootic was eliminated (due to the implemented efforts or spontaneously), and during the following two years no rabies cases in carnivores were registered in the Flagstaff area.

However, additional outbreaks caused by the same RABV variant occurred during 2004–2005: 6 striped skunks, 2 gray foxes, and a domestic cat (*Felis catus*), and during 2008–2009: 16 foxes, 1 skunk, and 1 ringtail cat (*Bassariscus astutus*). Extensive TVR programs for skunks and oral rabies vaccination (ORV) for foxes were implemented each time. Both outbreaks ceased, but the question about possible continuous perpetuation and evolution of bat RABV in populations of mesocarnivores re-arose with new magnitude.

The purpose of this study was to identify whether the Flagstaff outbreaks originated from a single introduction of bat RABV into mesocarnivores, with perpetuation in their populations, or each outbreak was caused by independent introduction of RABV. We also sought to identify whether the bat RABV variant, that caused Flagstaff outbreaks was different from other bat RABV variants, which had not caused such outbreaks in carnivores, and whether this RABV underwent recent genetic changes that might facilitate a host shift to carnivores (given that no similar outbreaks were documented in the Flagstaff area before 2001).

To answer these questions, we undertook a large-scale genetic investigation of bat RABVs, isolated in the Flagstaff area during 2001–2009 from carnivores, via sequencing of their complete genomes, evolutionary analysis, and comparison to the genomes of viruses isolated from big brown bats during the same time frame and historically. Furthermore, we included into genetic analysis representative RABVs from all major phylogenetic lineages circulating in North American bats and carnivores, and other complete and partial genomes from RABV variants circulating in other parts of the world.

## Materials and Methods

### Samples

Brain specimens from carnivores and bats from the Flagstaff area, where the presence of RABV antigens were demonstrated by the direct fluorescent antibody (DFA) test [18], were provided by the Arizona Department of Health Services, Bureau of State Laboratory Services, Phoenix, Arizona, USA, and the US Department of Agriculture (USDA)/Animal and Plant Health Inspection Service (APHIS)/Wildlife Services, Phoenix, Arizona, USA. At the Centers for Disease Control and Prevention (CDC), Atlanta, Georgia, USA, the samples were subjected to typing with antinucleocapsid monoclonal antibodies [13], and stored for further investigation at −80°C;. In total, we were able to collect brain specimens from 29 carnivores involved in the outbreaks caused by bat RABVs in the Flagstaff area during 2001–2009 (15 skunks from 2001; a skunk and a fox from 2004; a skunk and a domestic cat from 2005; 9 foxes and a ringtail cat from 2009). For two specimens that were unavailable in our study, N gene sequences were collected from GenBank where they were present from the previous investigation [17]. In addition, we obtained brain material from 9 big brown bats, infected with the same RABV variant, for the period of 2001–2010. Six historical big brown bat RABV isolates (obtained during 1975–1999), representing the same virus variant, and one isolate from a sister phylogenetic RABV lineage, were retrieved from CDC archives.

For comparative purposes, we included representatives of the major RABV lineages circulating in North America which had been sent to CDC via routine surveillance or in the framework of previous projects.

### Reverse-transcription polymerase chain reaction (RT-PCR) and gene sequencing

Total RNA was extracted from brain tissue using TRIZol reagent (Invitrogen, Carlsbad, CA, USA). Amplification of RABV genes and sequencing of the complete genomes was performed as described previously [19]. The primers are present in Table S2. The leader and trailer sequences were elucidated via RNA circularization, PCR-amplification across the ligated termini, and cloning of the PCR products [19]. For all carnivore specimens the complete genomes were generated from the primary field samples of brain. For several outbreak specimens which demonstrated over 99% nucleotide identity, the genome termini (parts of the leader and trailer sequences, which demonstrated 100% identity or 1–2 random nucleotide substitutions) were not generated, because this cumbersome operation was not expected to provide new valuable information.

For the majority of bat samples, limited amounts of brain tissues were available. Therefore, amplification and sequencing of the N, P, M and G genes was performed from the field samples, whereas amplification and sequencing of the L gene and the genome termini was performed after one intracerebral passage in suckling mice. The reliability of this approach was tested on 17 randomly selected samples, for which sequences of the G mRNA (about 2000 nucleotides) were determined from the field samples and after one intracerebral passage in suckling mice. No nucleotide substitutions were detected after the passage when sequences were compared. For the 7 historical big brown bat RABV isolates, two vampire bat isolates from Mexico (originating from cattle), and a white-nosed coati isolate from an outbreak documented in Mexico, the complete viral genomes were determined after one intracerebral passage in suckling mice, because no primary brain tissues were available.

**Figure 1:**
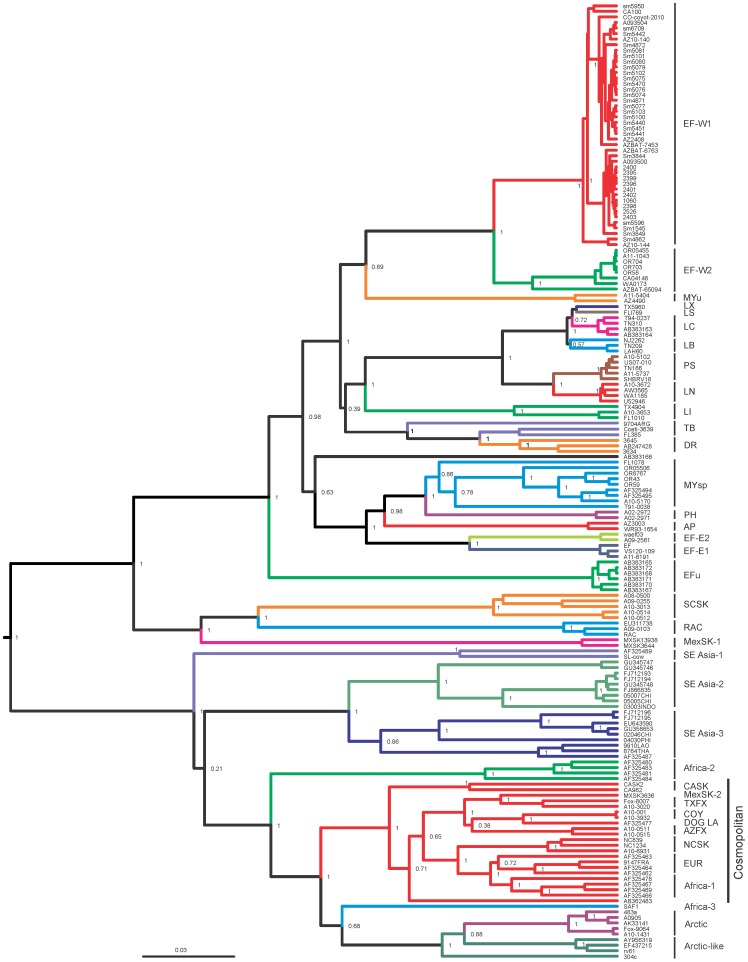
Bayesian tree of viruses, included in the study, based on the entire coding region of the G gene (1572 nuc). Lineage abbreviations: EF-W1 and EF-W2 – *Eptesicus fuscus*, with predominantly western distribution; MYu – *Myotis yumanensis*; LX – *Lasiurus xanthinus*; LS – *Lasiurus seminolus*; LC – *Lasiurus cinereus*; LB – *Lasiurus borealis*; PS – *Perimyotis subflavus*; LN – *Lasionycteris noctivagans*; LI – *Lasiurus intermedius*; TB – *Tadarida brasiliensis*; DR – *Desmodus rotundus*; MYsp – *Myotis* spp; PH – *Parastrellus hesperus*; AP – *Antrozous pallidus*; EF-E1 and EF-E2 – *Eptesicus fuscus*, with predominantly eastern and central distribution; EFu – *Eptesicus furinalis*; SCSK – south-central skunk; RAC – North-American Raccoon; MexSK-1 – Mexican skunk, variant 1; SE Asia 1, 2 and 3 – diverse dog RABV lineages circulating in the South-East Asia; Africa-2 – dog RABV lineage from the central and western Africa; CASK – California skunk; MexSK-2 – Mexican skunk, variant 2; TXFX – Texas gray fox; COY – coyote; DOG LA – dog RABV from Latin America; AZFX – Arizona gray fox; NCSK – north-central skunk; EUR – fox viruses from moderate latitudes of Eurasia; Aftrca-1 – dog RABV, broadly distributed in Africa; Africa-3 – mongoose RABV from southern Africa; Arctic – Arctic RABV from Eurasia and North America; Arctic-like – Arctic-like RABV from southern and eastern Asia.

In total, we sequenced 66 complete RABV genomes, and 20 additional genomes with truncated leader and trailer sequences. We complemented the dataset with representative genomes of RABV lineages circulating worldwide, retrieved from GenBank. Given the limited number of complete RABV genomes available in GenBank and our focus on the New World RABV lineages, we also included in the dataset a greater number of sequences of separate viral genes (with the complete coding region as the minimum requirement) from RABV lineages circulating in the New World, either available from GenBank or generated during our study. Information on the samples is provided in the Table S1 and Figure 1. The datasets included 168, 157, 103, 172 and 103 sequences for the N, P, M, G and L genes, respectively.

### Analysis of the genetic information

The assembly of viral genomes was performed either in MUSCLE [20] or in BioEdit, version 7.0.5 [21]. Translation of nucleotide sequences into deduced amino acid sequences, as well as calculation of identity values, was performed in BioEdit. Multiple alignments of complete genomes, separate genes, and non-coding genome regions were built by the ClustalW method, implemented in BioEdit and in ClustalX, version 2.0.17 [22]. The alignments were tested for recombinations using the Recombination Detection Program, version 2 [23].

### Phylogenetic analysis

Phylogenetic analysis was performed by the maximum likelihood method using PhyML, version 3.0 [24]. Statistical support of tree topology was derived from 500–1000 bootstrap replicates. Bayesian analyses were performed in MrBayes, version 3.2 [25] or in BEAST, version 1.3 [26]. Appropriate nucleotide substitution models were evaluated in MEGA, version 5.01 [27]. The general time-reversible model incorporating both invariant sites and a gamma distribution (GTR+I+G) was favored for all datasets. The base frequencies were estimated from the model, with the first two codon positions partitioned separately from codon position 3, and expansion population growth parameterized for growth rate was used as the coalescent tree prior. Two simultaneous runs, each with four Markov chains, were performed for 10,000,000 generations and sampled every 1,000 generations. The final inference of the tree was summarized from both runs with the initial 10% of samples discarded as burn-in. The trees were visualized using FigTree program, version 1.3.1 [26].

### Positive selection analysis

**Figure 2:**
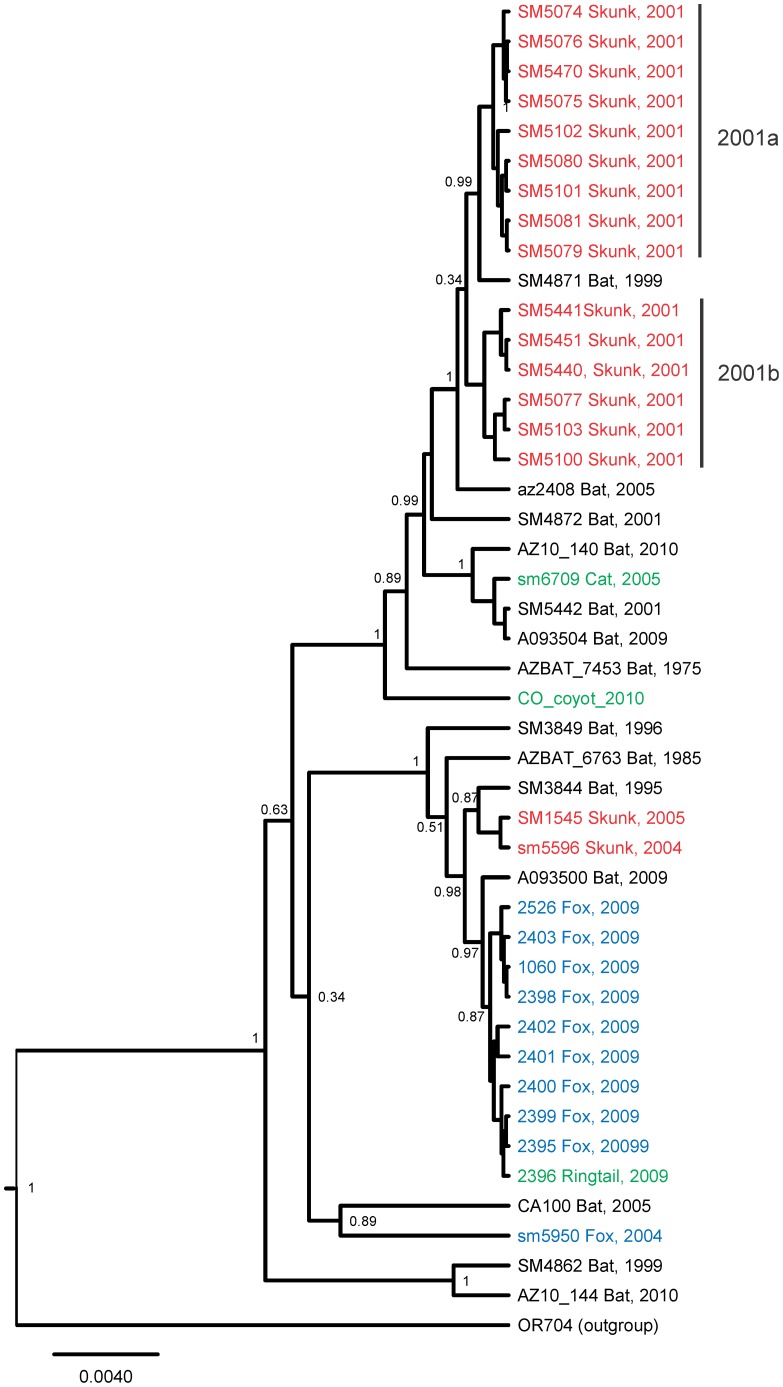
Bayesian tree of the EF-W1 lineage. Skunk viruses are colored in red, fox viruses are colored in blue, other mesocarnivoran viruses are colored in green; bat viruses are black. Posterior probabilities are indicated at key nodes.

**Figure 3:**
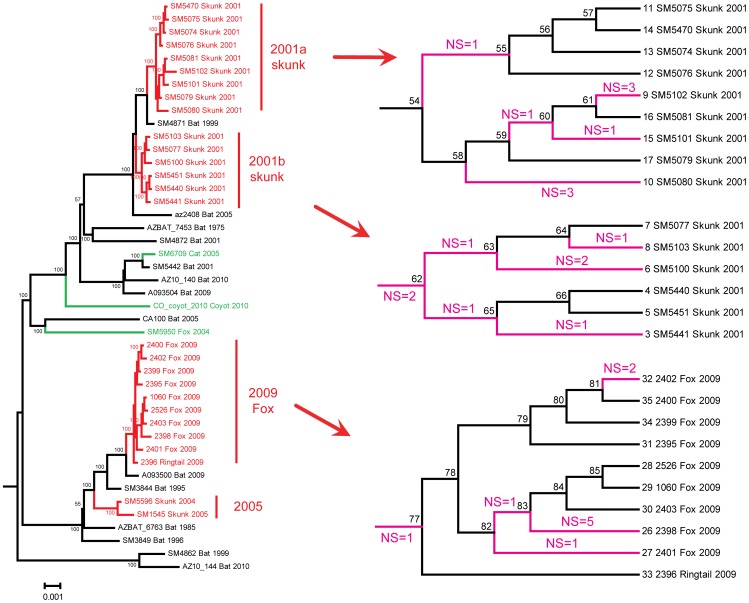
Reconstruction of post-host shift amino acid changes. Left: Bayesian tree of EF-W1 lineage, based on the entire coding region of G gene (1572 nuc). The branches involved in host-shift are marked with red. The amino acid changes occurred within these branches were defined “post-host shift” changes. Other branches that lead to terrestrial mammal RABV are marked with green. Right: the magnification of the three clusters involved in the Flagstaff host shifts. For clarity, the branch length did not reflect substitutions per site. The branches with non-synonymous changes are marked with pink, and numbers of changes are shown above and below. For more details see Table 2.

To examine if there was episodic positive selection following putative host shifts, we performed branch-specific selection analyses using the CODEML program implemented in the PAML package, version 4.4 [28]. In these analyses, branch(es) expected to be under positive selection were specified as “foreground” branch(es) while the rest were specified as “background” branches. We separated the ω (the ratios of non-synonymous to synonymous mutations, *d*N/*d*S) of foreground and background branches, and estimated these two parameters under a branch-specific model. Positive selection was indicated if the ω ratio was significantly higher in foreground branches than in background branches. The significance of the result was evaluated through a likelihood ratio test (LRT) against a null model assuming a single ω ratio for all branches. To test whether RABV underwent positive selection following a host shift from bats to carnivores, we specified the branches leading to outbreak clusters and branches within the outbreak clusters as foreground, and other branches within the EF-W1 lineage as background branches (Figures 2, 3). To account for potential difference of selection in different RABV genes, the tests were performed for each gene alignment separately.

### Convergent evolution analysis

We performed amino acid ancestral reconstructions to find convergent or parallel changes for different clusters or lineages of RABVs. Based on the obtained phylogeny (Figures 1, S1) and the five gene datasets, we estimated the most likely amino acids at all nodes using an empirical Bayes method (WAG+F as substitution model) implemented in PAML. The estimated amino acids at ancestral nodes were then used to infer the changes that occurred along all branches in the tree. A Perl script was used to identify convergent amino acid changes (i.e. changes from different amino acids that resulted in the same amino acid) and parallel amino acid changes (i.e. changes that start and result in the same amino acid) between branch pairs, both as potential signal for convergent evolution.

## Results

### General characterization of viral genomes sequenced in this study

The genomes varied in length from 11,899 nt to 11,930 nt. The shortest genome (vampire bat isolate 3645DR) had a long continuous deletion (23 nt) within the G-L intergenic region (the 3′ non-coding tail of the G mRNA), compared to the alignment of other RABV genomes. The second vampire RABV isolate in our study, 3634DR, did not have such a deletion.

Another feature was detected in the leader regions of several bat RABV genomes. All RABV genomes described to date harbored A in the position 10 of their leader regions (A_10_). Furthermore, the first 12 nt of their leader regions were strictly complementary to the terminal 12 nt of their trailer regions [29]. However, all but one available in our study RABV genomes, associated with lasiurine bats and *Perimyotis subflavus* (seven viruses from *Lasiurus* spp., one from *Lasionycteris noctivagans*, and one from *P. subflavus*) harbored G_10_ in their leader region (introducing non-complementarity) like it was documented previously in several non-RABV bat lyssaviruses [19], [30], [31]. The only virus from this cluster that did not have such a substitution was SHBRV-18. Besides this cluster, we documented G_10_ in the genome of RABV associated with the pallid bat (*Antrozous pallidus*).

Another feature was observed in one of the several groups of RABV, recovered from skunks during the Flagstaff outbreaks (Figure 2). All nine viruses from the cluster 2001a had insertion of an additional incomplete transcription termination and polyadenylation signal (TTP) between the regular TTP of the M gene and the standard intergene dinucleotide CT: GAAAAAAACAAAAAACT instead of GAAAAAAACT. This feature was not observed in other RABV genomes.

No evidence of recombination was observed in RABV genomes, generated during our study.

### Phylogenetic analysis

The ML and Bayesian phylogenetic analyses produced trees of identical topology for each gene dataset. The tree in the Figure 1 is based on the G gene sequences, as this dataset was most representative in our study, and it indicates that we incorporated into analysis representatives from all major RABV lineages, described elsewhere [1], [4], [5], [11], [12], [32].

The big brown bat viruses were segregated into two major clusters (EF-W, EF-E), with two lineages in each. The lineages EF-W1 and EF-W2 represented the viruses circulating predominantly in the western part of North America, whereas lineages EF-E1 and EF-E2 represented the viruses of eastern and central distribution. Geographic overlaps in distribution areas of these lineages occur, as was described, for example, in Colorado [14]. If compared to the recent publication on the distribution and dynamics of big brown bat RABV in Canada [33], our lineage EF-W2 corresponds to the lineage BB1 of that paper, whereas viruses from our lineage EF-W1 were not shown in that publication, perhaps because they are not present in Canada or their circulation is limited. Further, our lineage EF-E1 corresponds to the lineage BB5 (that also includes sub-lineages BB3 and BB4), and our lineage EF-E2 corresponds to the lineage BB2 from the same report [33].

All bat viruses that caused outbreaks in carnivores in the Flagstaff area during 2001–2009 belonged to the lineage EF-W1 (Figures 1, 2). The nucleotide identity of viral gene sequences derived from bats in this lineage was relatively high: 98.0–99.0%, 97.1–99.8%, 98.1–100%, 97.7–99.6% and 98.2–99.7% for N, P, M, G and L genes, respectively (amino acid identities 98.8–100%, 98.3–100%, 99.0–100%, 98.0–100% and 99.2–100%, respectively). Genomes of the oldest available isolates, such as AZBAT-7453 (1975) and AZBAT-6763 (1985) might be considered ancestral to minor sublineages, but in general fell within the diversity of viruses recovered from bats during 2001–2010.

The viruses recovered from skunks during 2001 were subdivided clearly into two lineages, 2001a and 2001b, based on the N, G, and L gene sequences. Despite the high identity values between the clades (99.5–99.8%), these clades were separated by 2 fixed synonymous substitutions within the N gene, 3 synonymous and 1 non-synonymous substitutions within the G gene, 7 synonymous and 1 non-synonymous substitutions within the L gene, and 2 substitutions within the G-L intergenic region. Moreover, all viruses from the lineage 2001a had the insertion of an additional incomplete TTP after the regular TTP of the M gene as described above. The bat virus SM4871 was more similar phylogenetically to the skunk lineage 2001a than to the skunk lineage 2001b (although it did not have the insertion of incomplete TTP after the M gene).

The P and M genes of viruses from clades 2001a, 2001b and from the bat SM4871 were identical, without any nucleotide substitutions. By comparison, the majority of viruses in each clade had 1–4 random nucleotide substitutions in the N, G, and L genes.

The outbreak isolates from 2004–2005 available in our study were not closely related to the viruses from 2001 or to each other, except two skunk samples, SM5596 and SM1545, which could have originated from one introduction of a bat RABV. The cat sample SM6709 was related to another group of bat viruses, and the fox sample SM5950 was different from all the above, but grouped with the bat sample CA100. Therefore, these 4 viruses represent at least 3 independent introductions of bat RABV into carnivores. In the absence of other outbreak samples from 2004–2005 we cannot establish whether any of these sequenced viruses could be a part of the major outbreak (potentially more introductions from bats could occur). Nevertheless, it is clear that neither of these 4 available viruses were related phylogenetically to the outbreaks that occurred during 2001 and 2008–2009.

The fox and ringtail cat viruses from 2009 constituted another monophyletic cluster of closely related viruses (identity values 99.7–100%, with the most conserved P and M genes, as was also observed in the skunk viruses from 2001, described above). The 2009 outbreak cluster was more similar phylogenetically to the bat virus A093500 than to the viruses recovered from previous outbreaks in carnivores.

A single case of spill-over infection in a coyote (*Canis latrans*) with a virus from the same lineage EF-W1 was reported from Colorado during 2010 (sample CO-coyot-2010; Figure 2).

### Positive selection analysis

Of the five genes tested for positive selection, two (M and L genes) were indicated to have significantly greater *d*N/*d*S following the host shift (i.e. at foreground branches) (Table 1). However, these positive signals were unlikely caused by adaptive evolution. In the M gene, the *d*N/*d*S ratio was estimated to be infinity within foreground branches. But the large value was due to the absence of synonymous changes rather than to excessive non-synonymous changes (only 1 non-synonymous change was observed). In the L gene, the greater *d*N/*d*S ratio was due to a transient (unfixed) amino acid polymorphism, because most of the non-synonymous changes were distributed at terminal branches rather than at internal branches (Table 1, Figure 3). Based on the ancestral reconstruction results, the ratio of non-synonymous mutation at terminal branches was 2 times greater than at internal branches. In one extreme case, 5 non-synonymous changes were observed in the branch leading to one virus, 2401 (Table 1). The overall *d*N/*d*S ratio dropped significantly at foreground branches after removing the sample 2401 from our dataset.

**Table 1:** Test results for positive selection analysis, and summary of amino acid substitutions.

Gene	LRT statistic^1^ (2*(Lha-Lh0))	Foreground *d*N/*d*S	Background *d*N/*d*S	Post host shift amino acid substitutions^3,4^^,^ 4
N	0.163	0.05521	0.03418	**3 A→T (60..15)**
P	0.000	0.06278	0.0001	none
M	6.989**	Inf^2^	0.077	173 M→I (76..77)
G	2.862	0.37652	0.14333	483 I→T (61..9), 464 A→S (52..62)
				**458 M→I (63..6), 348 G→R (65..3)**
				**273 V→L (81..32), 466 R→P (81..32)**
				**299 R→K (82..27)**
L	45.316**	0.64756	0.04838	2042 V→I (54..55), 2068 S→G (59..60)
				**235 G→V (61..9), 421 K→R (61..9)**
				**1818 L→S (58..10), 1820 I→K (58..10)**
				**1821 D→K (58..10)**, 1334 V→I (52..62)
				8 Y→H (62..63), **1332 S→T (64..8)**
				**263 P→L (63..6)**, 1882 A→V (62..65)
				1822 G→S (82..83), **1679 A→E (83..26)**
				**1685 K→R (83..26), 1689 I→V (83..26)**
				**1690 L→V (83..26), 1697 P→Q (83..26)**

1 Significance of test statistics (* *P*<0.05, ** *P*<0.01).

2 The infinite value is due to absence of synonymous change in foreground branch.

3 The information is arranged in the order: position, change, branch.

4 Amino acid changes with bold character were changes that occurred at terminal branches.

The ancestral reconstruction results also indicated that RABV sequences were highly similar before and after the host shifts. This observation can be extended to noncoding regions, where almost identical sequences were observed between host shift samples and background (bat) samples. In other words, except for a few stochastic changes, the virus sequences remained the same following the shift into another host.

### Convergent evolution analysis

No convergent or parallel changes were observed among the three independent host shift events (lineages 2001a, 2001b, and 2009; Figure 3), suggesting there was no detectable common mechanism that drove the adaptive evolution of viruses after each host shift. Therefore, we examined the hypothesis of pre-host shift adaptation. Under this hypothesis, we assumed that the ability to circulate in carnivores has been determined by neutral mutation in the RABV populations circulating in bats. We evaluated convergent and parallel changes between branches leading to EF-W lineages and those leading to carnivore-associated RABVs or bat RABVs associated with *Desmodus rotundus* (DR) and *Tadarida brasiliensis* (TB) (the latter involved into host shift in the white-nosed coatis [16]; Figure S1). Six parallel changes were observed between EF-W and carnivore RABV ancestral branches, whereas 3 parallel changes were observed between EF-W and DR&TB ancestral branches (Table 2). These sites detected to undergo convergent or parallel changes, except for site 464 in G gene, were all relatively conserved, with 2–5 substitutions across the entire phylogeny of RABV.

**Table 2:** Convergent changes among branches of interest in the bat and terrestrial RABV datasets.

Comparisons	Protein	Convergent changes	Branches involved^1^
Among host shift clusters	N	none	none
	P	none	none
	M	none	none
	G	none	none
	L	none	none
EF-W versus Terrestrial	N	56 I→V	127..128 and 103..184
		115 N→D	128..129 and 103..109
	P	48 S→N	127..128 and 103..184
	M	none	none
	G	196 K→R	127..128 and 103..184
		485 P→S	127..128 and 103..184
	L	1778 R→K	128..129 and 103..184
EF-W versus DR & TB	N	none	none
	P	none	none
	M	none	none
	G	242 S→T	127..128 and 112..122
		464 V→A	128..129 and 112..122
	L	107 H→Y	127..128 and 112..122

1 Branch numbers are corresponding to the number shown in the nodes (Figure S1).

In general, there were multiple fixed (conserved within specific lineages) amino acids in each protein that differentiated bat viruses from the “terrestrial” viruses, part of which were shared by viruses from the “bat-associated” RABVs from the raccoon (RAC), south-central skunk (SCSK), and one of Mexican skunk (MexSK-1) lineages. Based on the amino acid patterns, all viruses from the Flagstaff outbreaks were typical bat viruses. Moreover, no conservative substitutions were detected when historical bat isolates from the EF-W1 lineage (1975–1999) were compared to the recent isolates (2001–2010), either from bats or mesocarnivores. The number of substitutions was limited, and they all were randomly scattered among the sequences compared.

For the amino acid sites detected to undergo convergent or parallel changes, we reconstructed their evolutionary histories throughout the entire RABV tree. Although 6 parallel changes were observed between EF-W and carnivore RABV lineages, they were not specific between these two groups. For five of the sites detected, more than one bat RABV lineages shared the same convergence between EF-W and carnivore-associated RABV (Figure S3, A, B, C, D, and E), whereas no host shifts into mesocarnivores have been reported for those bat RABV lineages. One parallel change (P→S_485_ in the G) was unique for EF-W among all bats lineages. However, this site experienced multiple S→P substitutions within the carnivore RABV clades (Figure S3, F). On the other hand, the convergence between EF-W and DR&TB lineages involved three sites which did not overlap with carnivore RABVs. Among these, position 464 of the G was hyper-variable and thus was not likely to be involved in pre- shift adaptation (Figure S3, G); position 107 of the L had only two variants: H and Y, but the H variant was shared by both bat and carnivore RABV clades (Figure S3, H), and therefore this site is not likely to contribute to the adaptive differences of RABV between bats and terrestrial mammals. Only the substitution S→T_242_ in the G ectodomain merits additional attention (Figure S2). All but one representatives of carnivore RABV lineages, available in our study, have A_242_. These include such “bat-associated” carnivore RABV lineages as RAC and SCSK but exclude MexSK-1, which has T_242_. In contrast, the majority of bat viruses have S_242_. The exceptions include all big brown bat RABVs (viruses from lineages EF-E1 and EF-E2 have A_242_, whereas viruses from lineages EF-W1 and EF-W2 have T_242_). In addition, T_242_ was found in RABVs, associated with *T. brasiliensis* (lineage TB) and in several bat viruses from Brazil, predominantly associated with *Eptesicus furinalis* (lineage EFu; Figure S2).

Bat viruses with T_242_ have caused host shifts to carnivores more frequently than viruses with S_242_. These included 3 outbreaks in the Flagstaff area (or even 4, if we consider that viruses from the outbreak of 2004–2005, unavailable in our study, were the result of one introduction from bats); an outbreak in gray foxes in Oregon during 2010, that was caused by an introduction of a big brown bat RABV from lineage EF-W2 [34]; and an outbreak in coatis in Mexico during 2008 [16], caused by a bat virus from lineage TB. In contrast, viruses with S_242_, despite frequently detected spillover infections, caused only one documented outbreak in gray foxes in Oregon during 2009 [34], [35]; Figure S2.

## Discussion

One of the major questions we addressed in this study was whether bat RABV was re-introduced repeatedly into populations of mesocarnivores in the Flagstaff area during 2001–2009, or perpetuated in their populations after a single introduction in 2001. Our phylogenetic reconstructions demonstrated clearly that multiple introductions of bat viruses from the EF-W1 clade occurred into the skunk and fox populations independently. Moreover, the outbreak of 2001 most likely resulted from two virus incursions, each causing secondary transmission within the skunk population. The differences between viruses from these two clusters are supported by molecular and epidemiological data. Viruses from clade 2001a were collected during January–April from the northeastern part of the Flagstaff area, whereas viruses from clade 2001b were collected during March–July from the southern and western parts [17]. An alternative hypothesis, that each rabies case in carnivores could be an individual spill-over infection from a bat, was rejected as well because the sequence similarity of viruses within each outbreak cluster was much greater than the similarity between bat viruses from the EF-W1 lineage, and between viruses from different outbreaks.

Each host shift of bat viruses into carnivores in the Flagstaff outbreaks was transient, likely due to control efforts of state and federal services, including TVR programs for skunks and ORV for gray foxes. However, it is unknown what would have happened if no such significant efforts for outbreak containment had been implemented: whether outbreaks would self-eliminate, or the virus would perpetuate in populations of mesocarnivores. Virus circulation is a balanced equilibrium of multiple components, including genetic and antigenic properties of the pathogen, pathobiology of infection on the individual host level, and ecological properties of the host on the population level [3]. Although significant knowledge has been generated during a long history of studies on rabies in wildlife [36], [37], the evolutionary genetics and host shifts of RABV and other emerging viruses are poorly understood [12], [38].

Putative adaptation at the sequence level, if present, can take place at two stages of host shift. First, it may take place after cross-species transmission, for the virus to adjust to the new host environment (post-shift adaptation). Under this scenario, the virus may undergo a series of active changes in the genome under selective pressures to adapt to the new host, which can be reflected by higher *d*N/*d*S values or non-neutral convergent evolution. We performed positive selection analyses on branches that represent the post-host shift evolution. Although the test yielded positive results in M and L genes, they were most likely false positives. In fact, the EF-W1 viruses isolated from mesocarnivores during the Flagstaff outbreaks were closely related and could not be distinguished from those isolated from bats. Only a limited number of amino acid changes occurred at the post-shift stage, and these were found on terminal branches instead of on internal branches, indicating a lack of amino acid fixation after the host shift. Moreover, no convergence was detected in these amino acid changes among the three independent host shift events. Therefore, the RABVs in this study are unlikely to have undergone post-shift adaptation. As we established that each outbreak was caused by a separate virus introduction and continued only for several months, it is not surprising that adaptive changes in viral genomes did not accumulate during such a short time span.

We addressed whether viruses from the EF-W1 lineage that caused Flagstaff outbreaks were different from those circulating in bats historically. The phylogenetic analyses indicated that the historical bat isolates from the EF-W1 lineage were closely related to those causing multiple host-shift events, which suggested that viruses of this lineage have been capable of causing host shifts at least since 1975. Previous presumptive outbreaks in carnivores might have been missed because of the inherited limitations of the passive surveillance system. Alternatively, ecological factors may have favored outbreak conditions and multiple spill-over incidents during 2001, 2004–2005 and 2008–2009.

In general, a virus circulating in a certain reservoir host may already be competent for circulation in a new host (pre-shift adaptation). We examined this scenario by looking for neutral convergence between the EF-W and carnivore RABV lineages, and between the EF-W and DR+TB lineages. A number of sites were discovered to have converged between these lineages/clades. However, it is still unclear whether these convergences rendered a higher chance for successful host shift events. One amino acid substitution which can be considered potentially as a marker of pre-adaptation of bat RABV to cause effective infection in carnivores is the S→T_242_ in the G ectodomain. The amino acid in this position was significant for pathogenicity in the Nishigahara strain: the parental virus with A_242_ was pathogenic in a mouse model, whereas a mutant with S_242_ was attenuated [39]. Another recent study demonstrated that A_242_ increases virus spread between infected cells [40]. No studies on other amino acids in this position, including T_242_, have been described to date.

The RABV variants associated with carnivores have A_242_, and one lineage associated with Mexican skunks (MexSK-1) has T_242_. In contrast, the majority of bat RABV variants have S_242_. We know only one outbreak, caused by a virus with S_242_ (of a *Myotis* bat origin) in gray foxes in Oregon, during 2009 [35]. That outbreak was self-eliminating without implementation of any control actions. It is notable that lyssaviruses of other species (which all except Mokola virus are associated with bats) also have S_242_, and infect carnivores very infrequently [41]. This may suggest that lyssaviruses with S_242_ are less pathogenic to carnivores.

By comparison, bat viruses with T_242_ caused at least five outbreaks in carnivores, documented during the last decade, and were transmitted efficiently among skunks, foxes and coatis. However, the significance of T_242_ for the ability of bat viruses to switch to carnivores should be appreciated with caveats. For example, T_242_ is present in viruses of the TB lineage. Only one outbreak caused by a virus from this lineage was documented in coatis in Mexico [16]. However, RABV of the TB lineage is prevalent in large populations of *T. brasiliensis* bats in the southern US, but no outbreaks were documented in mesocarnivores from these areas, despite their frequent chances of exposure to rabid bats. Moreover, the viruses from EF-E1 and EF-E2 lineages broadly distributed in North America have A_242_, similarly to the viruses circulating in carnivores. Nevertheless, no outbreaks in carnivores caused by these viruses were documented in the US and Canada. Additional reverse genetics studies coupled with animal experiments in captivity are needed to elucidate the significance of T_242_ in the RABV G ectodomain for adaptation of bat viruses to circulation in carnivores.

In general, our study resolved several questions on the origin of Flagstaff rabies outbreaks, phylogenetic relationships of viruses involved in these outbreaks, and stability of their genomes over a limited period of time. Concurrently, our data raise questions on different aspects of RABV shifts from bats to carnivores. These include the significance of substitutions detected in viral genomes under different evolutionary models; likelihood of host shift to carnivores for different bat RABV variants; and outcomes of outbreaks in carnivores, caused by bat viruses, if no containment measures are implemented by humans.

In addition, we generated a significant dataset of complete and near-complete genomes of RABV from different lineages which warrant further extensive investigations. Our findings pose important questions on the molecular biology of RABV. For example, what is the significance of A→G_10_ substitution in the leader region, which previously had been observed only in several non-RABV bat lyssaviruses? This substitution and the following lack of complementarity may be important for virus replication, as the termini are involved in genome encapsidation and polymerase recognition [42]. It is remarkable that to date this substitution has been found only in genomes of bat lyssaviruses from different species [19], [30], [31]. On the other hand, this finding demonstrates inappropriateness of generation of “complete” viral genomes via RT-PCR with primers complementary to the genome termini, and substituting sequences of the real viral genome termini by sequences of the primers used for their amplification [43].

In this context, the termini of the SHBRV-18 genome should also be confirmed, as this was the only virus from the lasiurine RABV cluster that had A_10_ in the leader region. The SHBRV-18 was isolated from a human, presumably infected by *L. noctivagans*, and was subjected to a passage in suckling mice before genome sequencing [44]. Further phylogenetic refinement demonstrated that SHBRV-18 belongs to the RABV variant associated with *P. subflavus*, rather than with *L. noctivagans*. Potentially, alteration of the leader sequence could occur during these passages in heterologous models.

Another interesting observation was the insertion of an additional incomplete TTP after the regular TTP of the M gene in one cluster of viruses, recovered from skunks during an outbreak of 2001. Previously, similar insertions were observed in the non-coding region of the N gene, right before the regular TTP in several specimens of European bat lyssavirus, type 1 [45]. The significance of such insertions is unknown. Perhaps, they appear randomly in RABV genomes as a result of TTP duplication, but are eliminated during further virus passages because of redundancy. At least during the Flagstaff outbreak, such viruses were efficiently transmitted between skunks. These and other observations made in our study warrant further investigations on multiple levels, including evolutionary and functional studies.

The gene sequences, generated during this study, were deposited in GenBank, with accession numbers JQ685892-JQ686013.

## Supporting Information

Figure S1 The phylogenetic positions of the three sets of branches included in the convergent evolution analyses. In the tree, each node is labeled with a unique number so that the locations of the branches can be easily tracked.
(TIF)

Figure S2 Bayesian tree of bat RABV lineages, including available spill-over and outbreak viruses. Bat viruses are shown in black, host-shift viruses from mesocarnivores are shown in red, and spill-over viruses are shown in green. Amino acid in position 242 of the glycoprotein is indicated next to each lineage. Asterisks next to the viruses Coati-3639 and OR8767 indicate that these host-shift viruses were obtained from outbreaks (based on epidemiological data and limited N gene sequence comparisons) but only one isolate from each was available for extensive genome sequencing.
(TIF)

Figure S3 Phylogenetic mapping of evolutionary changes in: A - site 56; B - site 115 of N protein; C - site 48 of P protein; D - site 196 of G protein; E - site 1778 of L protein; F - site 485 of G protein; G - site 464 of G protein; H - site 107 of L protein.
(PDF)

Table S1 Viruses used in the present study.
(DOC)

Table S2 Primers used in the present study.
(DOC)
