# Supplementary figures and images for: Molecular Inferences Suggest Multiple Host Shifts of Rabies Viruses from Bats to Mesocarnivores in Arizona during 2001–2009
Source: PLoS Pathog. 2012 Jun 21;8(6):e1002786. doi: 10.1371/journal.ppat.1002786 (PMC3380930; doi:10.1371/journal.ppat.1002786)

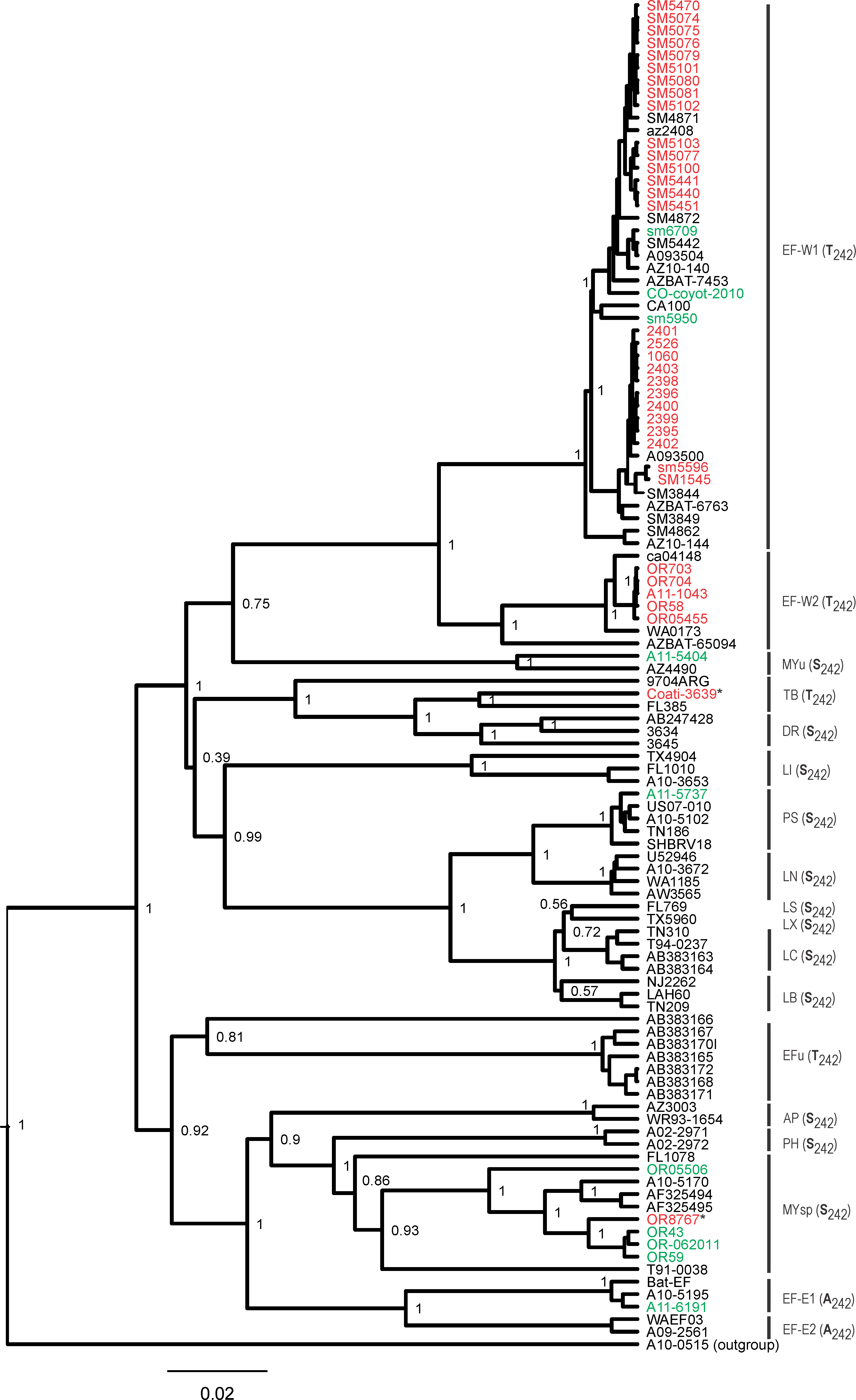

Supplement: Figure S2 — Bayesian tree of bat RABV lineages, including available spill-over and outbreak viruses. Bat viruses are shown in black, host-shift viruses from mesocarnivores are shown in red, and spill-over viruses are shown in green. Amino acid in position 242 of the glycoprotein is indicated next to each lineage. Asterisks next to the viruses Coati-3639 and OR8767 indicate that these host-shift viruses were obtained from outbreaks (based on epidemiological data and limited N gene sequence comparisons) but only one isolate from each was available for extensive genome sequencing. (TIF) [file ppat.1002786.s002.tif]

Figure S3A

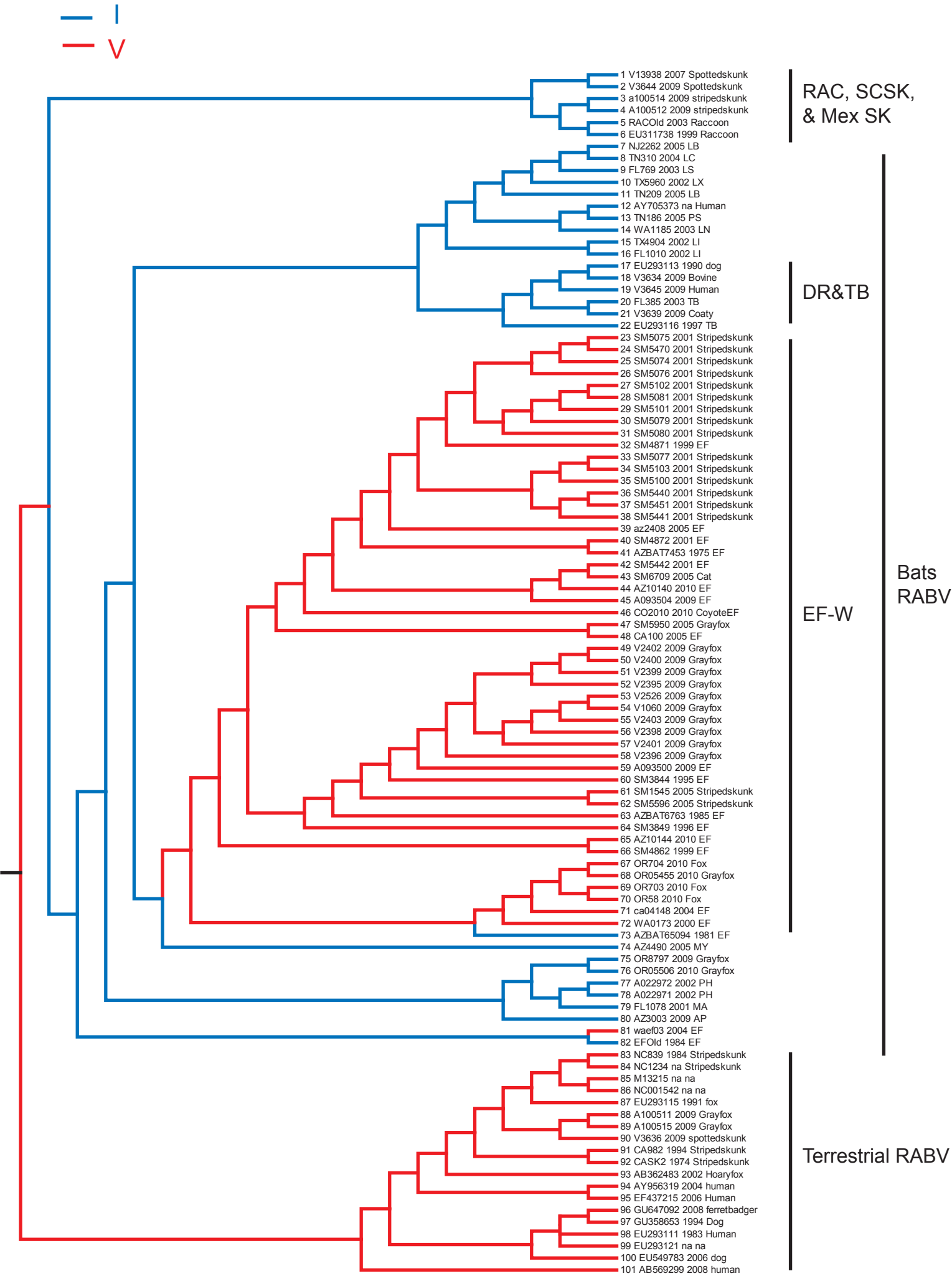

Figure S3B

— N  
— D  
— Others

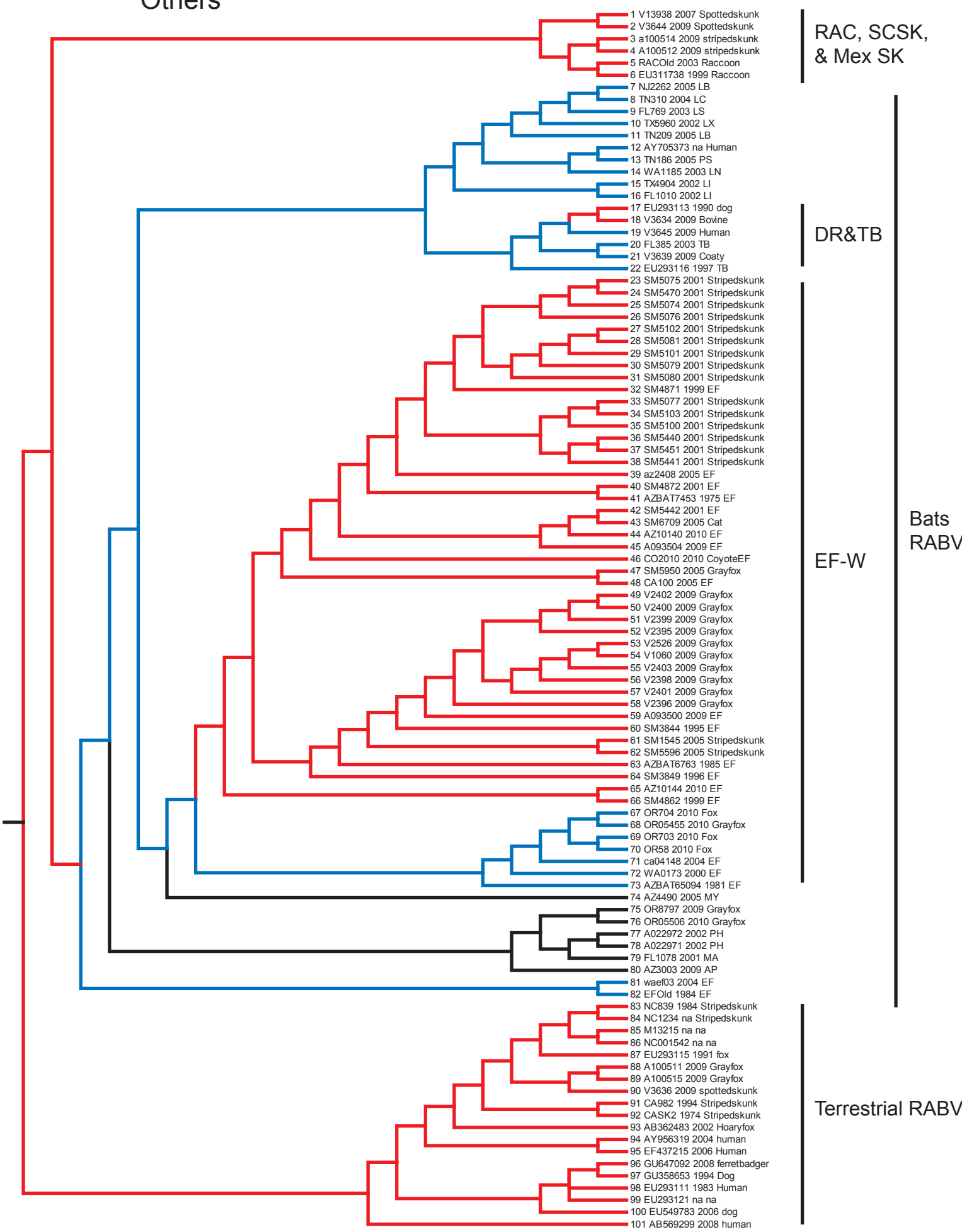

Figure S3C

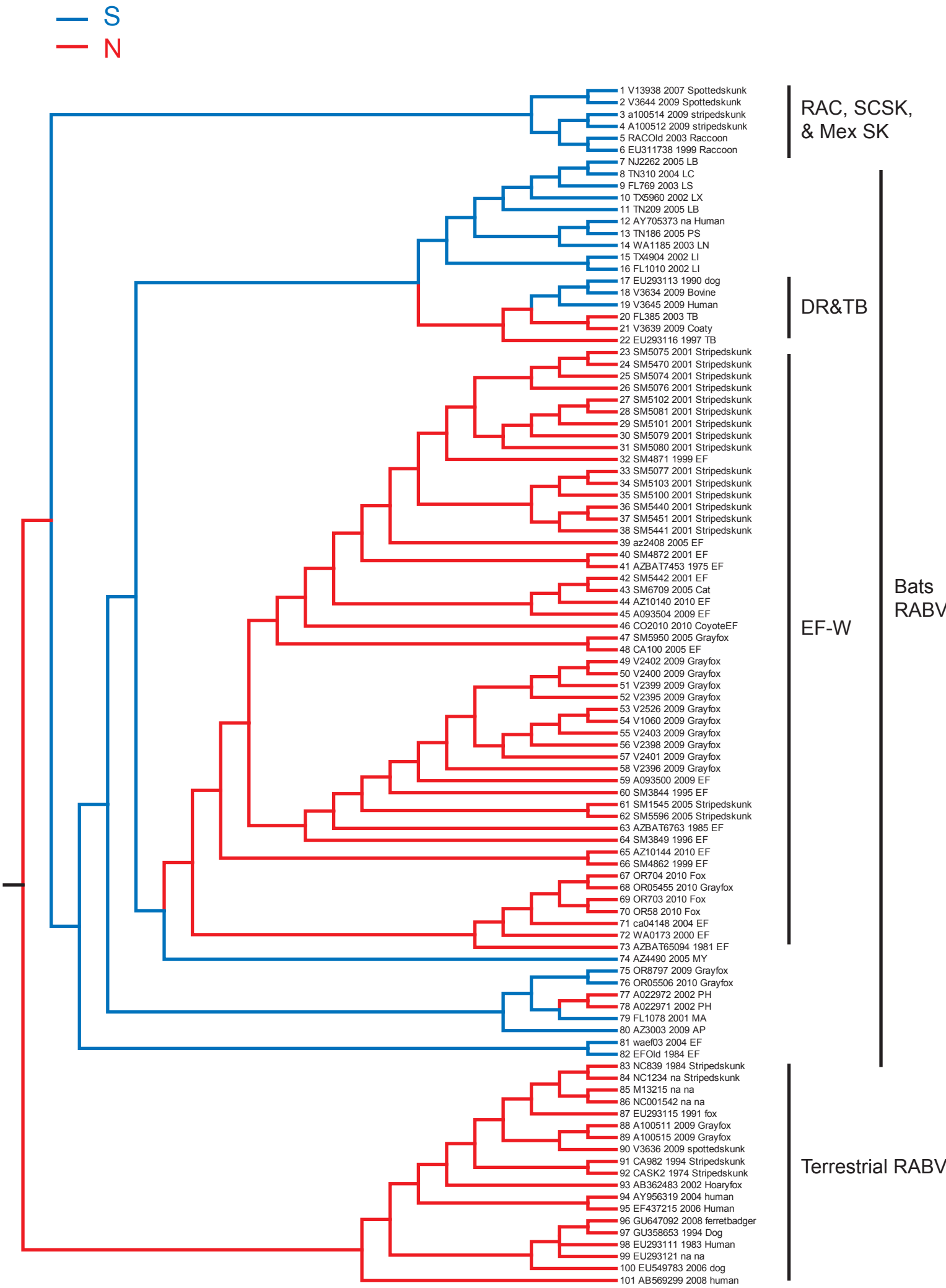

Figure S3D

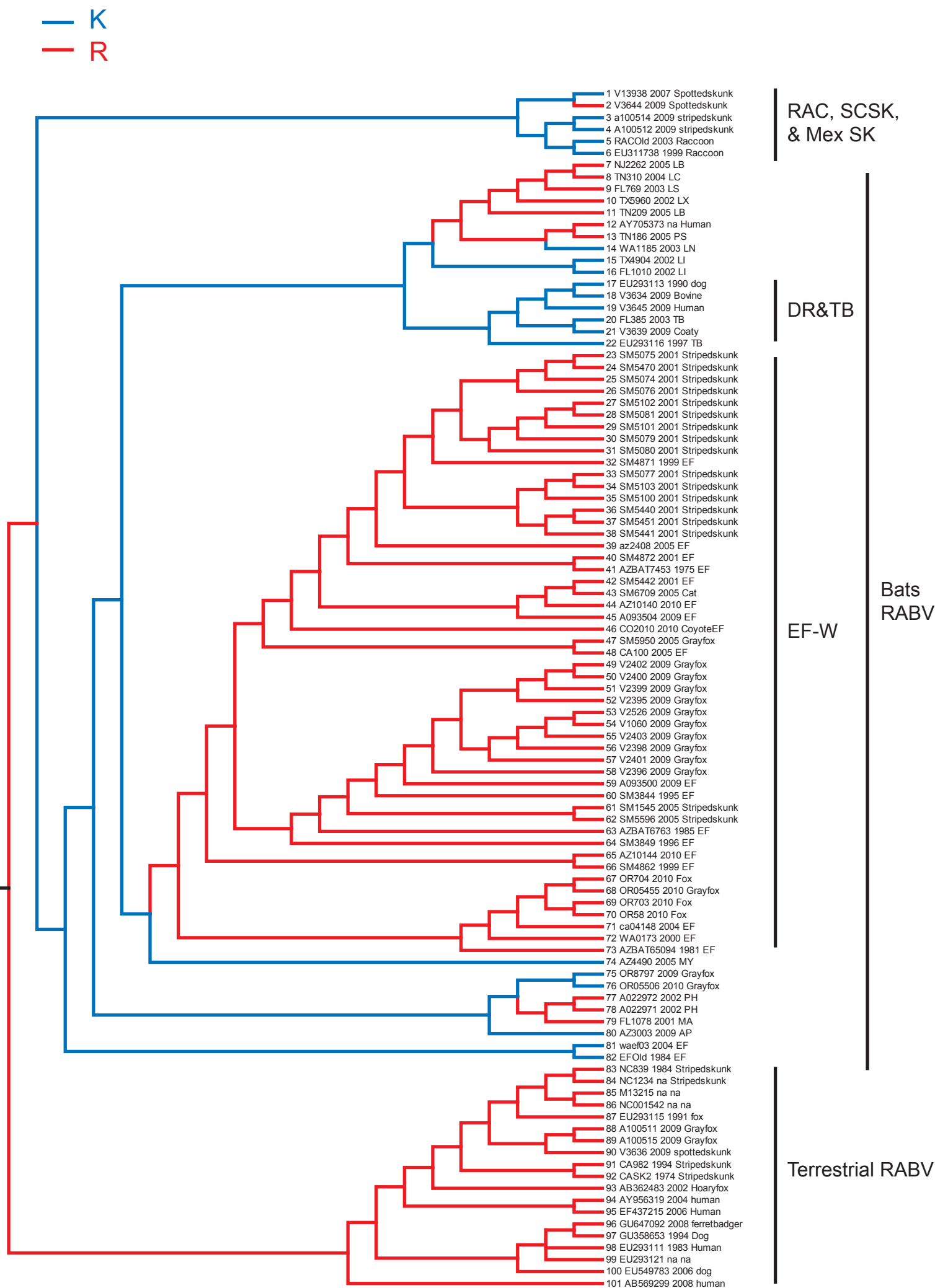

Figure S3E

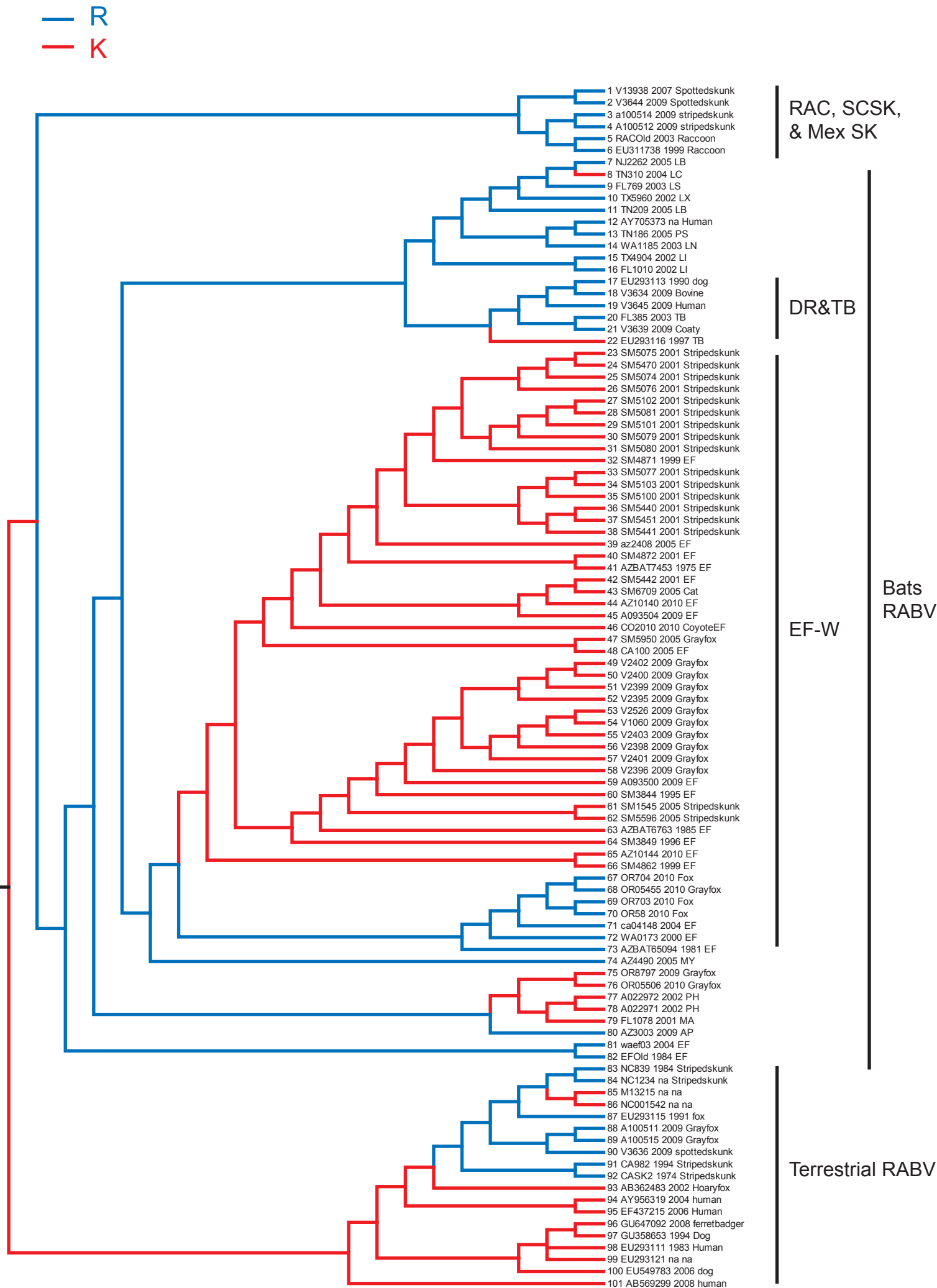

Figure S3F

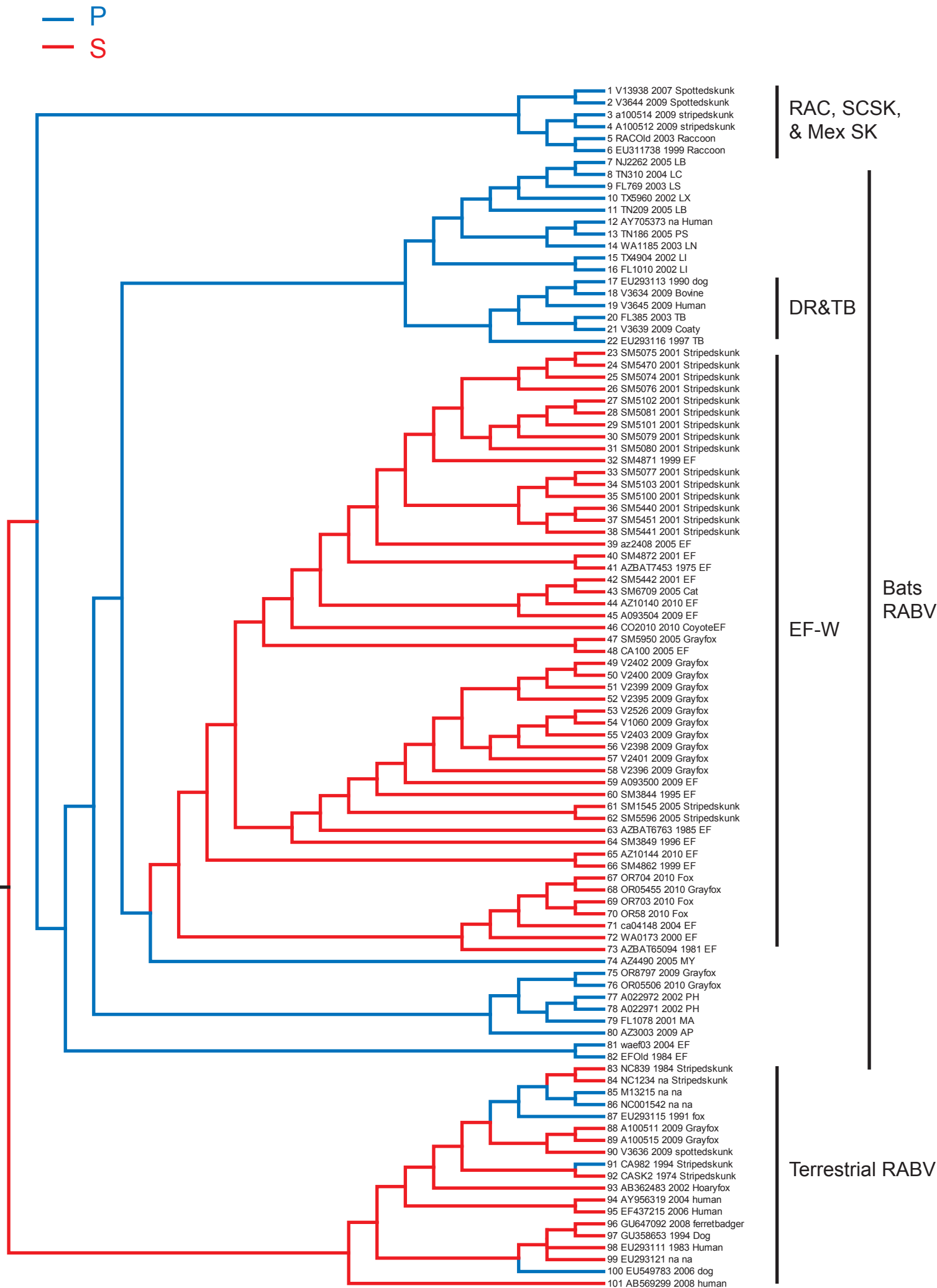

Figure S3G

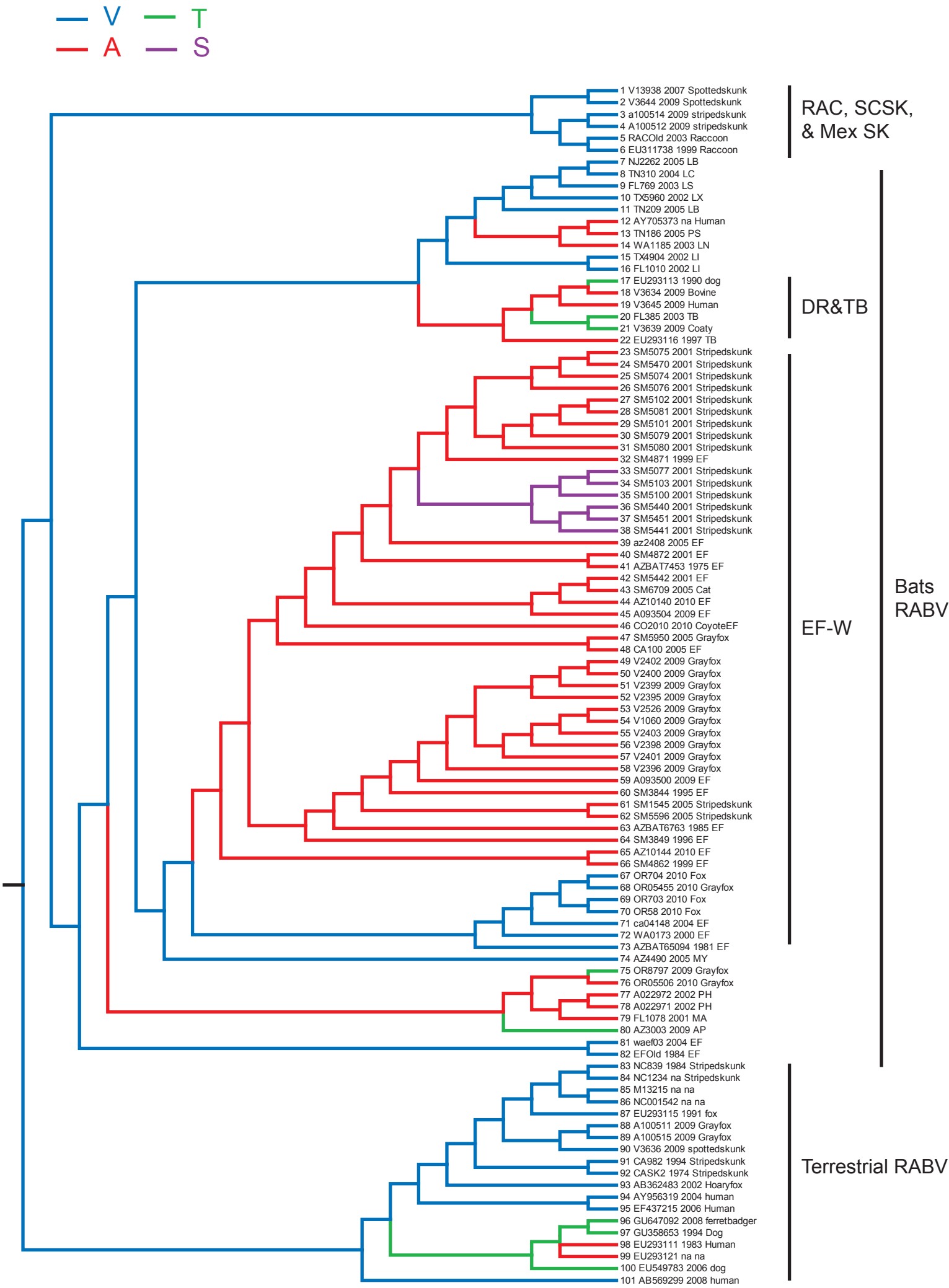

Figure S3H

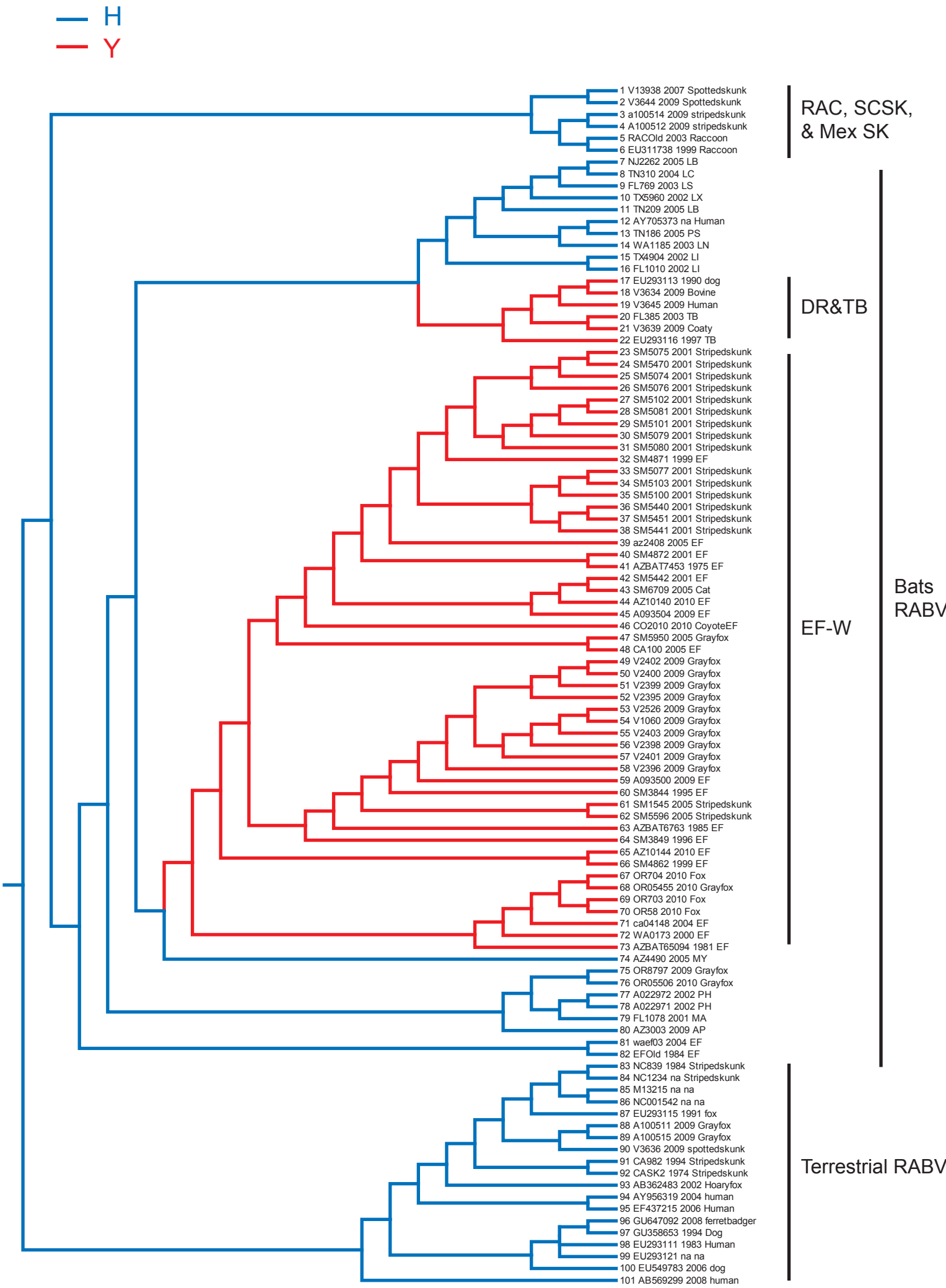

Supplement: Figure S3 — Phylogenetic mapping of evolutionary changes in: A - site 56; B - site 115 of N protein; C - site 48 of P protein; D - site 196 of G protein; E - site 1778 of L protein; F - site 485 of G protein; G - site 464 of G protein; H - site 107 of L protein. (PDF) [file ppat.1002786.s003.pdf]
