# Supplementary material for: Molecular Inferences Suggest Multiple Host Shifts of Rabies Viruses from Bats to Mesocarnivores in Arizona during 2001–2009
Source: PLoS Pathog. 2012 Jun 21;8(6):e1002786. doi: 10.1371/journal.ppat.1002786 (PMC3380930; doi:10.1371/journal.ppat.1002786)
Supplement: Table S1 — Viruses used in the present study. (DOC) [file ppat.1002786.s004.doc]

**Table S1**. Viruses used in the present study.

| **Virus name** | **Source animal** | **Phyligenetic clade** | **Year** | **Location** | **Genes sequenced** | **Sequence source** | **GenBank Aceesion No** | **Reference** |
| --- | --- | --- | --- | --- | --- | --- | --- | --- |
| SM5081 | *Mephitis mephitis* | EF-W1 | 2001 | USA, AZ/Flagstaff | Complete genome | Original brain | JQ685904 | This study |
| SM5080 | *Mephitis mephitis* | EF-W1 | 2001 | USA, AZ/Flagstaff | N, P, M, G, L | Original brain | JQ685927 | This study |
| SM5079 | *Mephitis mephitis* | EF-W1 | 2001 | USA, AZ/Flagstaff | Complete genome | Original brain | JQ685893 | This study |
| SM5077 | *Mephitis mephitis* | EF-W1 | 2001 | USA, AZ/Flagstaff | Complete genome | Original brain | JQ685911 | This study |
| SM5076 | *Mephitis mephitis* | EF-W1 | 2001 | USA, AZ/Flagstaff | Complete genome | Original brain | JQ685932 | This study |
| SM5074 | *Mephitis mephitis* | EF-W1 | 2001 | USA, AZ/Flagstaff | N, P, M, G, L | Original brain | JQ685935 | This study |
| SM4872 | *Eptesicus fuscus* | EF-W1 | 2001 | USA, AZ/Flagstaff | Complete genome | Original brain | JQ685960 | This study |
| SM1545 | *Mephitis mephitis* | EF-W1 | 2005 | USA, AZ/Flagstaff | Complete genome | Original brain | JQ685941 | This study |
| OR8767 | *Urocyon cinereoargenteus* | MYsp | 2009 | USA, OR | Complete genome | Original brain | JQ685957 | This study |
| CA100 | *Eptesicus fuscus* | EF-W1 | 2005 | USA, CA | Complete genome | N, P, M, G - original brain; L and termini - mouse | JQ685909 | This study |
| AZ2408 | *Eptesicus fuscus* | EF-W1 | 2005 | USA, AZ/Flagstaff | N, P, M, G, L | Original brain | JQ685926 | This study |
| A093504 | *Eptesicus fuscus* | EF-W1 | 2009 | USA, AZ/Flagstaff | Complete genome | N, P, M, G - original brain; L and termini - mouse | JQ685950 | This study |
| Coati -3639 | *Nasua narica* | TB | 2009 | Mexico, Cancun | Complete genome | Mouse | JQ685963 | This study |
| 2403 | *Urocyon cinereoargenteus* | EF-W1 | 2009 | USA, AZ/Flagstaff | N, P, M, G, L | Original brain | JQ685928 | This study |
| 2402 | *Urocyon cinereoargenteus* | EF-W1 | 2009 | USA, AZ/Flagstaff | N, P, M, G, L | Original brain | JQ685896 | This study |
| 2401 | *Urocyon cinereoargenteus* | EF-W1 | 2009 | USA, AZ/Flagstaff | Complete genome | Original brain | JQ685934 | This study |
| 2400 | *Urocyon cinereoargenteus* | EF-W1 | 2009 | USA, AZ/Flagstaff | N, P, M, G, L | Original brain | JQ685912 | This study |
| 2399 | *Urocyon cinereoargenteus* | EF-W1 | 2009 | USA, AZ/Flagstaff | N, P, M, G, L | Original brain | JQ685939 | This study |
| 2398 | *Urocyon cinereoargenteus* | EF-W1 | 2009 | USA, AZ/Flagstaff | N, P, M, G, L | Original brain | JQ685892 | This study |
| 2396 | *Bassariscus astutus* | EF-W1 | 2009 | USA, AZ/Flagstaff | N, P, M, G, L | Original brain | JQ685937 | This study |
| 2395 | *Urocyon cinereoargenteus* | EF-W1 | 2009 | USA, AZ/Flagstaff | N, P, M, G, L | Original brain | JQ685972 | This study |
| SM5075 | *Mephitis mephitis* | EF-W1 | 2001 | USA, AZ/Flagstaff | N, P, M, G, L | Original brain | JQ685949 | This study |
| 2526 | *Urocyon cinereoargenteus* | EF-W1 | 2009 | USA, AZ/Flagstaff | N, P, M, G, L | Original brain | JQ685976 | This study |
| 1060 | *Urocyon cinereoargenteus* | EF-W1 | 2009 | USA, AZ/Flagstaff | N, P, M, G, L | Original brain | JQ685908 | This study |
| A093500 | *Eptesicus fuscus* | EF-W1 | 2009 | USA, AZ/Flagstaff | Complete genome | N, P, M, G - original brain; L and termini - mouse | JQ685898 | This study |
| SM6709 | *Felis catus* | EF-W1 | 2005 | USA, AZ/Flagstaff | Complete genome | Original brain | JQ685945 | This study |
| SM5950 | *Urocyon cinereoargenteus* | EF-W1 | 2004 | USA, AZ/Flagstaff | Complete genome | Original brain | JQ685933 | This study |
| SM5596 | *Mephitis mephitis* | EF-W1 | 2004 | USA, AZ/Flagstaff | Complete genome | Original brain | JQ685964 | This study |
| SM5470 | *Mephitis mephitis* | EF-W1 | 2001 | USA, AZ/Flagstaff | Complete genome | Original brain | JQ685966 | This study |
| SM5451 | *Mephitis mephitis* | EF-W1 | 2001 | USA, AZ/Flagstaff | Complete genome | Original brain | JQ685959 | This study |
| SM5442 | *Eptesicus fuscus* | EF-W1 | 2001 | USA, AZ/Flagstaff | Complete genome | N, P, M, G - original brain; L and termini - mouse | JQ685897 | This study |
| SM5441 | *Mephitis mephitis* | EF-W1 | 2001 | USA, AZ/Flagstaff | Complete genome | Original brain | JQ685962 | This study |
| SM5440 | *Mephitis mephitis* | EF-W1 | 2001 | USA, AZ/Flagstaff | Complete genome | Original brain | JQ685969 | This study |
| SM5103 | *Mephitis mephitis* | EF-W1 | 2001 | USA, AZ/Flagstaff | N, P, M, G, L | Original brain | JQ685930 | This study |
| SM5102 | *Mephitis mephitis* | EF-W1 | 2001 | USA, AZ/Flagstaff | N, P, M, G, L | Original brain | JQ685906 | This study |
| SM5101 | *Mephitis mephitis* | EF-W1 | 2001 | USA, AZ/Flagstaff | Complete genome | Original brain | JQ685958 | This study |
| SM5100 | *Mephitis mephitis* | EF-W1 | 2001 | USA, AZ/Flagstaff | Complete genome | Original brain | JQ685940 | This study |
| SM5450 | *Eptesicus fuscus* | EF-W1 | 2001 | AZ/Flagstaff | N | Original brain | AY170413 | [1] |
| CO-Coyot-2010 | *Canis latrans* | EF-W1 | 2010 | USA, CO | Complete genome | Original brain | JQ685917 | This study |
| OR05455 | *Urocyon cinereoargenteus* | EF-W2 | 2010 | USA, OR | Complete genome | Original brain | JQ685948 | This study |
| OR05506 | *Urocyon cinereoargenteus* | MYsp | 2010 | USA, OR | Complete genome | Original brain | JQ685918 | This study |
| MXSK3636 | *Spilogale putorius* | Cosmopolitan (MexSK-2) | 2009 | Mexico | Complete genome | Mouse | JQ685975 | This study |
| RAC | *Procyon lotor* | RAC | 2003 | GA | Complete genome | Mouse | JQ685901 | This study |
| NC1234 | *Mephitis mephitis* | Cosmopolitan (NCSK) | ? | ? | Complete genome | Original brain | JQ685967 | This study |
| NC839 | *Mephitis mephitis* | Cosmopolitan (NCSK) | 1984 | USA, TN | Complete genome | Original brain | JQ685944 | This study |
| MXSK3644 | *Spilogale putorius* | MexSK-1 | 2009 | Mexico | Complete genome | Mouse | JQ685929 | This study |
| CASK2 | *Mephitis mephitis* | Cosmopolitan (CASK) | 1974 | USA, CA | Complete genome | Original brain | JQ685970 | This study |
| CA982 | *Mephitis mephitis* | Cosmopolitan (CASK) | 1994 | USA, CA | Complete genome | Original brain | JQ685894 | This study |
| A10-0515 | *Urocyon cinereoargenteus* | Cosmopolitan (AZFX) | 2009 | USA, AZ/Flagstaff | Complete genome | Original brain | JQ685899 | This study |
| A10-0514 | *Mephitis mephitis* | SCSK | 2009 | USA, AZ/Flagstaff | Complete genome | Original brain | JQ685938 | This study |
| A10-0512 | *Mephitis mephitis* | SCSK | 2009 | USA, AZ/Flagstaff | Complete genome | Original brain | JQ685968 | This study |
| A10-0511 | *Urocyon cinereoargenteus* | Cosmopolitan (AZFX) | 2009 | USA, AZ/Flagstaff | Complete genome | Original brain | JQ685943 | This study |
| MXSK13938 | *Spilogale putorius* | MexSK-1 | 2007 | Mexico | Complete genome | Mouse | JQ685954 | This study |
| FJ712194 | *Canis familiaris* | SE Aasia-2 | 2008 | China | Complete genome | ? | FJ712194 | [2] |
| GU345746 | *Canis familiaris* | SE Aasia-2 | 1992 | China | Complete genome | ? | GU345746 | Meng et al., unpubliched |
| 8764THA | *Homo sapiens* | SE Asia - 3 | 1983 | Thailand | Complete genome | ? | EU293111 | [3] |
| GU345748 | *Canis familiaris* | SE Aasia-2 | 2006 | China | Complete genome | ? | GU345748 | Meng et al., unpubliched |
| FJ712195 | *Melogale moschata* | SE Asia - 3 | 2008 | China | Complete genome | ? | FJ712195 | [2] |
| 9147FRA | *Vulpes vulpes* | Cosmopolitan (EUR) | 1991 | France | Complete genome | ? | EU293115 | [3] |
| FJ712196 | *Melogale moschata* | SE Asia - 3 | 2008 | China | Complete genome | ? | FJ712196 | [2] |
| GU358653 | *Canis familiaris* | SE Asia - 3 | 1994 | China | Complete genome | ? | GU358653 | Li et al., unpublished |
| GU345747 | *Homo sapiens* | SE Asia -2 | 1986 | China | Complete genome | ? | GU345747 | Meng et al., unpubliched |
| EU311738 | *Procyon lotor* | RAC | 1999 | Canada, ON | Complete genome | Original brain | EU311738 | [4] |
| EF437215 | *Homo sapiens* | Arctic-like | 2006 | India | Complete genome | ? | EF437215 | Desai et al., unpublished |
| rv61 | *Homo sapiens* | Arctic-like | 1987 | UK (via India) | N, G | Mouse | AY352493; JQ685981 | [5]; this study |
| 483a | *Vulpes lagopus* | Arctic | 1986 | Russia, Yakutia | N, G | Mouse | AY352487; JQ685982 | [5]; this study |
| 304c | *Vulpes corsac* | Arctic-like | 1977 | Russia, Tyva | N, G | Mouse | AY352459; JQ685983 | [5]; this study |
| RV1596 | *Vulpes vulpes* | Cosmopolitan (EUR) | 1987 | Russia, Pskov | N | Mouse | AY352474 | [5] |
| U43432 | *Nyctereutes procyonoides* | Cosmopolitan (EUR) | 1991 | Estonia | N | ? | U43432 | [6] |
| U42706 | *Vulpes vulpes* | Cosmopolitan (EUR) | 1986 | Yugoslavia | N | ? | U42706 | []6 |
| U22481 | *Vulpes vulpes* | Cosmopolitan (EUR) | 1987 | Saudi Arabia | N | ? | U22481 | [7] |
| U22480 | *Vulpes vulpes* | Cosmopolitan (EUR) | 1990 | Oman | N | ? | U22480 | [7] |
| U22483 | *Canis lupus* | Cosmopolitan (EUR) | 1987 | Iran | N | ? | U22483 | [7] |
| U42703 | *Vulpes vulpes* | Cosmopolitan (EUR) | 1976 | Yugoslavia | N | ? | U42703 | [6] |
| 9224TAN | *Canis familiaris* | Cosmopolitan (Africa - 1) | 1992 | Tanzania | N | ? | U22648 | [7] |
| 8670NGA | *Homo sapiens* | Cosmopolitan (Africa - 1) | 1983 | Nigeria | N | ? | U22488 | [7] |
| 8807ETH | *Crocuta crocuta* | Cosmopolitan (Africa - 1) | 1987 | Ethiopia | N | ? | U22637 | [7] |
| 8660GUI | *Canis familiaris* | Africa-2 | 1986 | Guinea | N | ? | U22487 | [7] |
| 8636HAV | *Canis familiaris* | Africa-2 | 1986 | Burkina Faso | N | ? | EU853614 | [7] |
| AB041966 | *Canis familiaris* | SE Asia - 1 | 1996 | Sri Lanka | N | ? | AB041966 | [8] |
| AY138549 | *Canis familiaris* | SE Asia - 1 | 1986 | Sri Lanka | N | ? | AY138549 | [9] |
| EU086205 | *Canis familiaris* | SE Asia - 3 | 2004 | Philippines | N | ? | EU086205 | [10] |
| AF467949 | *Cynictis penicillata* | Africa - 3 |  | S. Africa | N | ? | AF467949 | Jacob et al., unpublished |
| A0905 | *Canis familiaris* | Arctic | 2006 | USA, AK | N, G | Original brain | EF611853; JQ685980 | [11]; this study |
| FJ866835 | *Canis familiaris* | SE Asia - 2 | 2008 | China | Complete genome | ? | FJ866835 | Zhang et al., unpublished |
| EU643590 | *Homo sapiens* | SE Asia - 3 | 2006 | China | Complete genome | ? | EU643590 | [12] |
| AB362483 | *Lycalopex vetulus* | Cosmopolitan |  | Brazil | Complete genome | ? | AB362483 | [13] |
| AY956319 | *Homo sapiens* | Arctic-like | 2004 | Germany (via India) | Complete genome | ? | AY956319 | Pfefferle et al., unpublished |
| WA1185 | *Lasionycteris noctivagans* | LN | 2003 | USA, WA | Complete genome | N, P, M, G - original brain; L and termini - mouse | JQ685895 | This study |
| WA0173 | *Eptesicus fuscus* | EF-W2 | 2000 | USA, WA | Complete genome | N, P, M, G - original brain; L and termini - mouse | JQ685931 | This study |
| TX5960 | *Lasiurus xanthinus* | LX | 2002 | USA, TX | Complete genome | N, P, M, G - original brain; L and termini - mouse | JQ685910 | This study |
| TX4904 | *Lasiurus intermedius* | LI | 2002 | USA, TX | Complete genome | N, P, M, G - original brain; L and termini - mouse | JQ685915 | This study |
| TN310 | *Lasiurus cinereus* | LC | 2004 | USA, TN | Complete genome | N, P, M, G - original brain; L and termini - mouse | JQ685947 | This study |
| TN209 | *Lasiurus borealis* | LB | 2005 | USA, TN | Complete genome | N, P, M, G - original brain; L and termini - mouse | JQ685902 | This study |
| TN186 | *Perimyotis subflavus* | PS | 2005 | USA, TN | Complete genome | N, P, M, G - original brain; L and termini - mouse | JQ685922 | This study |
| NJ2262 | *Lasiurus borealis* | LB | 2005 | USA, NJ | Complete genome | N, P, M, G - original brain; L and termini - mouse | JQ685919 | This study |
| FL1078 | *Myotis austroriparius* | MYsp | 2001 | USA, FL | Complete genome | N, P, M, G - original brain; L and termini - mouse | JQ685921 | This study |
| FL769 | *Lasiurus seminolus* | LS | 2003 | USA, FL | Complete genome | N, P, M, G - original brain; L and termini - mouse | JQ685900 | This study |
| FL385 | *Tadarida brasiliensis* | TB | 2003 | USA, FL | Complete genome | N, P, M, G - original brain; L and termini - mouse | JQ685905 | This study |
| EF | *Eptesicus fuscus* | EF-E1 | 1984 | USA, PA | Complete genome | mouse | JQ685920 | This study |
| ca04148 | *Eptesicus fuscus* | EF-W2 | 2004 | USA, CA | Complete genome | N, P, M, G - original brain; L and termini - mouse | JQ685903 | This study |
| AZ4490 | *Myotis yumanensis* | My | 2005 | USA, AZ | Complete genome | N, P, M, G - original brain; L and termini - mouse | JQ685955 | This study |
| AZ3003 | *Antrozous pallidus* | AP | 2009 | USA, AZ | Complete genome | N, P, M, G - original brain; L and termini - mouse | JQ685971 | This study |
| waef03 | *Eptesicus fuscus* | EF-E2 | 2004 | USA, WA | Complete genome | N, P, M, G - original brain; L and termini - mouse | JQ685925 | This study |
| FL1010 | *Lasiurus intermedius* | LI | 2002 | USA, FL | Complete genome | N, P, M, G - original brain; L and termini - mouse | JQ685916 | This study |
| 3645DR | *Homo sapiens* | DR | 2009 | Mexico | Complete genome | mouse | JQ685953 | This study |
| SHBRV18 | *Homo sapiens* | PS |  | USA | Complete genome | mouse | AY705373 | [14] |
| 9704ARG | *Tadarida brasilensis* | TB | 1997 | Argentina | Complete genome | ? | EU293116 | [3] |
| EF40 | *Eptesicus fuscus* | EF-E1 | 1995 | Canada, ON | N | ? | AF351862 | [15] |
| EF3 | *Eptesicus fuscus* | EF-E2 | 1972 | Canada, ON | N | ? | AF351861 | [15] |
| wa1086 | *Eptesicus fuscus* | EF-W2 | 2003 | USA, WA | N | Original brain | GU644851 | [16] |
| wa267 | *Eptesicus fuscus* | EF-W2 | 2000 | USA, WA | N | Original brain | GU644682 | [16] |
| wa1043 | *Eptesicus fuscus* | EF-W2 | 2003 | USA, WA | N | Original brain | GU644683 | [16] |
| ML4 | *Myotis lucifugus* | MYsp | 1992 | Canada, BC | N | ? | AF351839 | [15] |
| ML6 | *Myotis lucifugus* | MYsp | 1994 | Canada, NS | N | ? | AF351838 | [15] |
| MC2 | *Myotis californicus* | MYsp | 1992 | Canada, BC | N | ? | AF351836 | [15] |
| ME1 | *Myotis evotis* | MYsp | 1992 | Canada, BC | N | ? | AF351835 | [15] |
| IB-Ch | *Tadarida* sp | TB | 1988 | Chile | N | ? | AF351850 | [15] |
| TB1 | *Tadarida brasiliensis* | TB | ? | USA, TX | N | ? | AF351849 | [15] |
| EF22 | *Eptesicus fuscus* | EF-W2 | 1988 | Canada, BC | N | ? | AF351830 | [15] |
| 804 | *Eptesicus fuscus* | EF-W2 | 1987 | USA, CA | N | ? | AF394887 | [17] |
| EF72 | *Eptesicus fuscus* | EF-E1 | 1998 | USA, CT | N | ? | AF351854 | [15] |
| EF57 | *Eptesicus fuscus* | EF-E1 | 1997 | Canada, ONT | N | ? | AF351859 | [15] |
| EF71 | *Eptesicus fuscus* | EF-E1 | 1998 | USA, CT | N | ? | AF351860 | [15] |
| 136 | *Eptesicus fuscus* | EF-E1 | 1984 | USA, RA | N | ? | AY039226 | [17] |
| EF34 | *Eptesicus fuscus* | EF-E2 | 1972 | Canada, BC | N | ? | AF351832 | [15] |
| 906 | *Eptesicus fuscus* | EF-E2 | 1986 | USA, WA | N | ? | AY039227 | [17] |
| EF31 | *Eptesicus fuscus* | EF-E2 | 1989 | Canada, SASK | N | ? | AF351831 | [15] |
| EF3 | *Eptesicus fuscus* | EF-E2 | 1972 | Canada, ONT | N | ? | AF351861 | [15] |
| 132 | *Eptesicus fuscus* | EF-E2 | 1972 | USA, PA | N | ? | AY039229 | [17] |
| EF19 | *Eptesicus fuscus* | EF-E2 | 1991 | Canada, ALTA | N | ? | AF351855 | [15] |
| 3634DR | Bovine | DR | 2009 | Mexico | N, P, M, G, L | Mouse | JQ685936 | This study |
| A02-2972 | *Parastrellus hesperos* | PH | 2002 | USA, AZ | Complete genome | N, P, M, G - original brain; L and termini - mouse | JQ685965 | This study |
| A02-2971 | *Parastrellus hesperos* | PH | 2002 | USA, CA | Complete genome | N, P, M, G - original brain; L and termini - mouse | JQ685952 | This study |
| AZBAT-7453 | *Eptesicus fuscus* | EF-W1 | 1975 | USA, AZ | Complete genome | Mouse | JQ685956 | This study |
| AZBAT-6763 | *Eptesicus fuscus* | EF-W1 | 1985 | USA, AZ | Complete genome | Mouse | JQ685913 | This study |
| AZBAT-65094 | *Eptesicus fuscus* | EF-W2 | 1981 | USA, AZ | Complete genome | Mouse | JQ685942 | This study |
| SM4862 | *Eptesicus fuscus* | EF-W1 | 1999 | USA, AZ/Flagstaff | Complete genome | N, P, M, G - original brain; L and termini - mouse | JQ685946 | This study |
| SM4871 | *Eptesicus fuscus* | EF-W1 | 1999 | USA, AZ/Flagstaff | Complete genome | N, P, M, G - original brain; L and termini - mouse | JQ685923 | This study |
| SM3844 | *Eptesicus fuscus* | EF-W1 | 1995 | USA, AZ/Flagstaff | Complete genome | N, P, M, G - original brain; L and termini - mouse | JQ685974 | This study |
| SM3849 | *Eptesicus fuscus* | EF-W1 | 1996 | USA, AZ/Flagstaff | Complete genome | N, P, M, G - original brain; L and termini - mouse | JQ685907 | This study |
| OR58 | *Urocyon cinereoargenteus* | EF-W2 | 2010 | USA, OR | N, P, M, G, L | Original brain | JQ685977 | This study |
| OR703 | *Urocyon cinereoargenteus* | EF-W2 | 2010 | USA, OR | N, P, M, G, L | Original brain | JQ685914 | This study |
| OR704 | *Urocyon cinereoargenteus* | EF-W2 | 2010 | USA, OR | N, P, M, G, L | Original brain | JQ685924 | This study |
| OR43 | *Urocyon cinereoargenteus* | MYsp | 2000 | USA, OR | N, G | Original brain | JQ685979; JQ686013 | This study |
| OR59 | *Urocyon cinereoargenteus* | MYsp | 2006 | USA, OR | N, G | Original brain | JQ685978; JQ686012 | This study |
| A11-1043 | *Canis latrans* | EF-W2 | 2011 | USA, OR | Complete genome | Original brain | JQ685973 | This study |
| AZ10-140 | *Eptesicus fuscus* | EF-W1 | 2010 | AZ/Flagstaff | Complete genome | N, P, M, G - original brain; L and termini - mouse | JQ685961 | This study |
| AZ10-144 | *Eptesicus fuscus* | EF-W1 | 2010 | AZ/Flagstaff | N, P, M, G, L | N, P, M, G - original brain; L and termini - mouse | JQ685951 | This study |
| 1741WC.SK | *Mephitis mephitis* | Cosmopolitan (NCSK) | 1992 | Canada | P | ? | AF369286 | [18] |
| 867WC.SK | *Mephitis mephitis* | Cosmopolitan (NCSK) | 1992 | Canada | P | ? | AF369285 | [18] |
| 9499.MYO | *Vulpes vulpes* | MYsp | 1993 | Canada | P | ? | AF369353 | [18] |
| B1.DG | *Canis familiaris* | Cosmopolitan (DOG-LA) | 1993 | Brazil | P | ? | AF369314 | [18] |
| I15.DG | *Canis familiaris* | SE Asia - 1 | 1995 | India | P | ? | AF369309 | [18] |
| Ir5.DG | *Canis familiaris* | Cosmopolitan (EUR) | 1993 | Iran | P | ? | AF369311 | [18] |
| ONT1.RFX | *Mephitis mephitis* | Arctic | 1991 | Canada | P | ? | AF369265 | [18] |
| ONT2.RFX | *Vulpes vulpes* | Arctic | 1990 | Canada | P | ? | AF369266 | [18] |
| P1.DG | *Canis familiaris* | Cosmopolitan (DOG-LA) | 1994 | Paraguay | P | ? | AF369315 | [18] |
| RU7.FX | *Vulpes vulpes* | Cosmopolitan (EUR) | 1988 | Kazakhstan | P | ? | AF369274 | [18] |
| RU9.RD | *Nyctereutes procyonoides* | Arctic-like | 1988 | Russia | P | ? | AF369284 | [18] |
| V027.DG | *Canis familiaris* | Cosmopolitan (Africa - 1) | 1988 | Tunissia | P | ? | AF369322 | [18] |
| V034.RFX | *Vulpes vulpes* | Cosmopolitan (EUR) | 1987 | Switzerland | P | ? | AF369272 | [18] |
| V046.MG | *Cynictis penicillata* | Africa - 3 | 1990 | S. Africa | P | ? | AF369298 | [18] |
| V050.MG | *Cynictis penicillata* | Africa - 3 | 1990 | S. Africa | P | ? | AF369301 | [18] |
| V113.DG | *Canis familiaris* | SE Asia - 1 | 1986 | Sri Lanka | P | ? | AF369320 | [18] |
| V118.DG | *Canis familiaris* | SE Asia - 1 | 1986 | Sri Lanka | P | ? | AF369321 | [18] |
| V121.DG | *Canis familiaris* | Arctic-like | 1989 | Nepal | P | ? | AF369317 | [18] |
| V211.SK | *Mephitis mephitis* | SCSK | 1994 | USA, TX | P | ? | AF369287 | [18] |
| V213.SK | *Mephitis mephitis* | Cosmopolitan (COY) | 1994 | USA, TX | P | ? | AF369289 | [18] |
| V216.SK | *Mephitis mephitis* | SCSK | 1994 | USA, TX | P | ? | AF369291 | [18] |
| V217.CO | *Canis latrans* | Cosmopolitan (COY) | 1994 | USA, TX | P | ? | AF369337 | [18] |
| V250.CD | *Canis familiaris* | Cosmopolitan (Africa - 1) | 1994 | S. Africa | P | ? | AF369297 | [18] |
| V264.MG | *Cynictis penicillata* | Africa - 3 | 1995 | S. Africa | P | ? | AF369302 | [18] |
| V285.RFX | *Vulpes vulpes* | Cosmopolitan (EUR) | 1992 | Check Republic | P | ? | AF369273 | [18] |
| V461.DG | *Canis familiaris* | Africa - 2 | 1996 | Nigeria | P | ? | AF369326 | [18] |
| V466.DG | *Canis familiaris* | Cosmopolitan (DOG-LA) | 1996 | Nigeria | P | ? | AF369329 | [18] |
| V660.FX | *Vulpes vulpes* | Cosmopolitan (EUR) | ? | Israel | P | ? | AF369280 | [18] |
| V667.DG | *Canis familiaris* | Cosmopolitan (Africa - 1) | ? | Ethiopia | P | ? | AF369331 | [18] |
| V670.DG | *Canis familiaris* | Cosmopolitan (Africa - 1) | ? | Tanzania | P | ? | AF369333 | [18] |
| V458.DG | Bovine | Arctic-like | 1991 | India | P | ? | AF369330 | [18] |
| 058.BBB | *Eptesicus fuscus* | EF-E1 | 1993 | Canada | P | ? | AF369338 | [18] |
| 2994.BBB | *Eptesicus fuscus* | EF-E2 | 1993 | Canada | P | ? | AF369339 | [18] |
| 3694.MYO | *Myotis* sp | MYsp | 1996 | Canada | P | ? | AF369349 | [18] |
| 4398.SHB | *Lasionycteris noctivagans* | LN | 1980 | Canada | P | ? | AF369345 | [18] |
| 4805.BBB | *Eptesicus fuscus* | EF-E2 | 1995 | Canada | P | ? | AF369350 | [18] |
| 4887.LBB | *Myotis lucifugus* | MYsp | 1994 | Canada | P | ? | AF369344 | [18] |
| 6832.RB | *Lasiurus borealis* | LB | 1991 | Canada | P | ? | AF369351 | [18] |
| 7890.LBB | *Myotis lucifugus* | MYsp | 1997 | Canada | P | ? | AF369352 | [18] |
| V077.SHB | *Lasionycteris noctivagans* | LN | 1988 | Canada | P | ? | AF369346 | [18] |
| V078.BBB | *Eptesicus fuscus* | EF-W2 | 1988 | Canada | P | ? | AF369341 | [18] |
| V084.BBB | *Eptesicus fuscus* | EF-E2 | 1989 | Canada | P | ? | AF369340 | [18] |
| V089.LBB | *Myotis lucifugus* | MYsp | 1992 | Canada | P | ? | AF369343 | [18] |
| V102.MYO | *Myotis evotis* | MYsp | 1992 | Canada | P | ? | AF369354 | [18] |
| V103.HB | *Lasiurus cinereus* | LC | 1992 | Canada | P | ? | AF369347 | [18] |
| V151.BBB | *Eptesicus fuscus* | EF-E2 | 1989 | Canada | P | ? | AF369355 | [18] |
| V158.SHB | *Lasionycteris noctivagans* | LN | 1991 | Canada | P | ? | AF369356 | [18] |
| V170.BBB | *Eptesicus fuscus* | EF-E2 | 1993 | Canada | P | ? | AF369357 | [18] |
| V179.LBB | *Myotis lucifugus* | MYsp | 1993 | Canada | P | ? | AF369358 | [18] |
| V230.BBB | *Eptesicus fuscus* | EF-W1 | 1994 | USA, TX | P | ? | AF369342 | [18] |
| V231.RB | *Lasiurus borealis* | LB | 1994 | USA, TX | P | ? | AF369348 | [18] |
| V235.FTB | *Tadarida brasiliensis* | TB | 1994 | USA, TX | P | ? | AF369359 | [18] |
| FL.RAC | *Procyon lotor* | RAC | 1987 | USA, FL | P | ? | AF369294 | [18] |
| NY.RAC | *Procyon lotor* | RAC | 1992 | USA, NY | P | ? | AF369293 | [18] |
| A10-3020 | *Urocyon cinereoargenteus* | Cosmopolitan (TXFX) | 2008 | TX | G | Original brain | JQ685984 | This study |
| A10-3932 | *Urocyon cinereoargenteus* | Cosmopolitan (COY) | 2010 | AZ | G | Original brain | JQ685985 | This study |
| A10-3013 | *Mephitis mephitis* | SCSK | 2009 | TX | G | Original brain | JQ685986 | This study |
| A10-6931 | *Vulpes vulpes* | Cosmopolitan (NCSK) |  | MN | G | Original brain | JQ685987 | This study |
| A10-1431 | *Vulpes vulpes* | Arctic |  | NH | G | Original brain | JQ685988 | This study |
| A10-001 | *Urocyon cinereoargenteus* | Cosmopolitan (COY) | 2010 | AZ | G | Original brain | JQ685989 | This study |
| Fox-9064 | *Vulpes vulpes* | Arctic |  | NH | G | Original brain | JQ685990 | This study |
| A09-0255 | *Canis lupus* | SCSK | 2009 | OK | G | Original brain | JQ685991 | This study |
| SL-cow | Bovine | SE Asia - 1 |  | Sri Lanka | G | Original brain | JQ685992 | This study |
| A08-0500 | *Felis catus* | SCSK | 2008 | CO | G | Original brain | JQ685993 | This study |
| A09-0103 | *Canis latrans* | RAC | 2009 | AL | G | Original brain | JQ685994 | This study |
| Fox-8007 | *Urocyon cinereoargenteus* | Cosmopolitan (TXFX) | 2008 | TX | G | Original brain | JQ685995 | This study |
| YUG1-WF | Bovine | Cosmopolitan (EUR) | 1984 | Yugoslavia | G | ? | AF325463 | [19] |
| HUN1-HM | *Homo sapiens* | Cosmopolitan (EUR) | 1992 | Hungary | G | ? | AF325462 | [19] |
| POL1-RD | *Nyctereutes procyonoides* | Cosmopolitan (EUR) | 1985 | Poland | G | ? | AF325464 | [19] |
| MOR3-HM | *Homo sapiens* | Cosmopolitan (Africa -1 ) | 1990 | Morocco | G | ? | AF325469 | [19] |
| TUN1-HM | *Homo sapiens* | Cosmopolitan (Africa -1 ) | 1986 | Tunissia | G | ? | AF325466 | [19] |
| MEX1-DG | *Canis familiaris* | Cosmopolitan (DOG LA) | 1991 | Mexico | G | ? | AF325477 | [19] |
| SAF1-MG | *Cynictis penicillata* | Africa - 3 | 1987 | S. Africa | G | ? | AF325485 | [19] |
| NIG1-DG | *Canis familiaris* | Africa - 2 | 1990 | Niger | G | ? | AF325480 | [19] |
| MAU1-CL | *Camelus dromedarius* | Africa - 2 | 1986 | Mauritania | G | ? | AF325483 | [19] |
| CAM1-UN | *Canis familiaris* | Africa - 2 | 1988 | Cameroon | G | ? | AF325481 | [19] |
| GUI1-DG | *Canis familiaris* | Africa - 2 | 1986 | Guinea | G | ? | AF325484 | [19] |
| MAL1-HM | *Homo sapiens* | SE Asia - 3 | 1985 | Malaysia | G | ? | AF325487 | [19] |
| NEP1-DG | *Canis familiaris* | SE Asia - 1 | 1989 | Nepal | G | ? | AF325489 | [19] |
| 03003INDO | *Canis familiaris* | SE Asia - 2 | 2003 | Indonesia | G | ? | EU086151 | [10] |
| 05007CHI | *Canis familiaris* | SE Asia - 2 | 2004 | China | G | ? | EU086148 | [10] |
| 05005CHI | *Canis familiaris* | SE Asia - 2 | 2005 | China | G | ? | EU086146 | [10] |
| 02046CHI | *Canis familiaris* | SE Asia - 3 | 1994 | China | G | ? | EU086144 | [10] |
| 04030PHI | *Homo sapiens* | SE Asia - 3 | 2004 | Philippines | G | ? | EU086155 | [10] |
| 9910LAO | *Canis familiaris* | SE Asia - 3 | 1999 | Laos | G | ? | EU086152 | [10] |
| MOR2-DG | *Canis familiaris* | Cosmopolitan (Africa -1 ) | 1987 | Marocco | G | ? | AF325467 | [19] |
| MAD1-DG | *Canis familiaris* | Cosmopolitan (Africa -1 ) | 1985 | Madagascar | G | ? | AF325478 | [19] |
| AB247428 | *Desmodus rotundus* | DR | ? | Brazil | G | ? | AB247428 | Sato, unpublished |
| AB383164 | *Non-hematophagous bat* | LC | ? | Brazil | G | ? | AB383164 | Sato, unpublished |
| AB383163 | *Non-hematophagous bat* | LC | ? | Brazil | G | ? | AB383163 | Sato, unpublished |
| AB383165 | *Non-hematophagous bat* | EFu | ? | Brazil | G | ? | AB383165 | [20] |
| AB383166 | *Non-hematophagous bat* |  | ? | Brazil | G | ? | AB383166 | [20] |
| AB383167 | *Eumops auripendulus* | EFu | ? | Brazil | G | ? | AB383167 | [20] |
| AB383168 | *Eptesicus furinalis* | EFu | ? | Brazil | G | ? | AB383168 | [20] |
| AB383170 | *Eptesicus furinalis* | EFu | ? | Brazil | G | ? | AB383170 | [20] |
| AB383171 | *Eptesicus furinalis* | EFu | ? | Brazil | G | ? | AB383171 | [20] |
| AB383172 | *Nyctinomops laticaudatus* | EFu | ? | Brazil | G | ? | AB383172 | [20] |
| USA8-BT | *Myotis* sp | MYsp | 1981 | USA, MT | G | ? | AF325494 | [19] |
| USA9-BT | *Myotis* sp | MYsp | 1982 | USA, MT | G | ? | AF325495 | [19] |
| U52946 | *Homo sapiens* | LN | 1994 | USA, CA | G | ? | U52946 | [21] |
| A09-2561 | *Eptesicus fuscus* | EF-E2 | 2010 | USA, IA | G | Original brain | JQ685996 | This study |
| A10-5170 | *Myotis* sp | MYsp | 2010 | USA, ID | G | Original brain | JQ685997 | This study |
| T91-0038 | *Myotis* sp | MYsp | 1991 | USA, CA | G | Original brain | JQ685998 | This study |
| T94-0237 | *Lasiurus cinereus* | LC | 1994 | USA, CA | G | Original brain | JQ685999 | This study |
| US07-010 | *Perimyotis subflavus* | PS | 2010 | USA, NC | G | Original brain | JQ686000 | This study |
| AW3565 | *Lasionycteris noctivagans* | LN | 2010 | USA, AL | G | Original brain | JQ686001 | This study |
| VS120-109 | *Eptesicus fuscus* | EF-E1 | 2010 | USA, NC | G | Original brain | JQ686002 | This study |
| A10-5102 | *Perimyotis subflavus* | PS | 2010 | USA, NC | G | Original brain | JQ686003 | This study |
| A10-3672 | *Lasionycteris noctivagans* | LN | 2009 | USA, AL | G | Original brain | JQ686004 | This study |
| A10-3653 | *Lasiurus intermedius* | LI | 2008 | USA, FL | G | Original brain | JQ686005 | This study |
| WR93-1654 | *Antrozous pallidus* | AP | 1993 | USA, AZ | G | Original brain | JQ686006 | This study |
| LAH60 | *Lasiurus borealis* | LB | 2010 | USA, NC | G | Original brain | JQ686007 | This study |
| OR-062011 | *Urocyon cinereoargenteus* | MYsp | 2011 | USA, OR | G | Original brain | JQ686011 | This study |
| A11-5404 | *Urocyon cinereoargenteus* | MYsp | 2011 | USA, AZ | G | Original brain | JQ686008 | This study |
| A11-5737 | *Vulpes vulpes* | PS | 2011 | USA, VA | G | Original brain | JQ686009 | This study |
| A11-6191 | Fox (unidentified) | EF-E1 | 2011 | USA, WV | G | Original brain | JQ686010 | This study |

**References for Table S1**

1. Leslie MJ, Messenger S, Rohde RE, Smith J, Cheshier R, et al. (2006) Bat-associated rabies virus in Skunks. Emerg Infect Dis 12: 1274-1277.

2. Lei YL, Wang XG, Tao XY, Li H, Meng SL, et al. (2010) Sequencing and analysis of complete genome of rabies viruses isolated from Chinese Ferret-Badger and dog in Zhejiang province. Bing Du Xue Bao 26: 45-52.

3. Delmas O, Holmes EC, Talbi C, Larrous F, Dacheux Let al. (2008) Genomic diversity and evolution of the lyssaviruses. PLoS One 3: e2057.

4. Szanto AG, Nadin-Davis SA, White BN (2008) Complete genome sequence of a raccoon rabies virus isolate. Virus Res 136: 130-139.

5. Kuzmin IV, Botvinkin AD, McElhinney LM, Smith JS, Orciari LA, et al. (2004) Molecular epidemiology of terrestrial rabies in the former Soviet Union. J Wildl Dis 40: 617-631.

6. Bourhy H, Kissi B, Audry L, Smreczak M, Sadkowska-Todys M, et al. (1999) Ecology and evolution of rabies virus in Europe. J Gen Virol. 80: 2545-2457.

7. Kissi B, Tordo N, Bourhy H (1995) Genetic polymorphism in the rabies virus nucleoprotein gene. Virology 209: 526-537.

8. Arai YT, Takahashi H, Kameoka Y, Shiino T, Wimalaratne O, et al. (2001) Characterization of Sri Lanka rabies virus isolates using nucleotide sequence analysis of nucleoprotein gene. Acta Virol 45: 327-333.

9. Nanayakkara S, Smith JS, Rupprecht CE (2003) Rabies in Sri Lanka: splendid isolation. Emerg Infect Dis 9: 368-371.

10. Bourhy H, Reynes JM, Dunham EJ, Dacheux L, Larrous F, et al. (2008) The origin and phylogeography of dog rabies virus. J Gen Virol 89: 2673-2681.

11. Kuzmin IV, Hughes GJ, Botvinkin AD, Gribencha SG, Rupprecht CE (2008) Arctic and Arctic-like rabies viruses: distribution, phylogeny and evolutionary history. Epidemiol Infect 136: 509-519.

12. Ming P, Du J, Tang Q, Yan J, Nadin-Davis SA, et al. (2009) Molecular characterization of the complete genome of a street rabies virus isolated in China. Virus Res 143: 6-14.

13. Mochizuki N, Kobayashi Y, Sato G, Itou T, Gomes AA, et al. (2009) Complete genome analysis of a rabies virus isolate from Brazilian wild fox. Arch Virol 154: 1475-1488.

14. Faber M, Pulmanausahakul R, Nagao K, Prosniak M, Rice AB, et al. (2004) Identification of viral genomic elements responsible for rabies virus neuroinvasiveness. Proc Natl Acad Sci U S A 101: 16328–16332.

15. Nadin-Davis SA, Huang W, Armstrong J, Casey GA, Bahloul C, et al. (2001) Antigenic and genetic divergence of rabies viruses from bat species indigenous to Canada. Virus Res 74: 139-156.

16. Streicker DG, Turmelle AS, Vonhof MJ, Kuzmin IV, McCracken GF, et al. (2010) Host phylogeny constrains cross-species emergence and establishment of rabies virus in bats. Science 329: 676-679.

17. Rohde RE, Mayes BC, Smith JS, Neill SU (2004) Bat rabies, Texas, 1996-2000. Emerg Infect Dis 10: 948-952.

18. Nadin-Davis SA, Abdel-Malik M, Armstrong J, Wandeler AI (2002) Lyssavirus P gene characterisation provides insights into the phylogeny of the genus and identifies structural similarities and diversity within the encoded phosphoprotein. Virology 298: 286-305.

19. Badrane H, Tordo N (2001) Host switching in Lyssavirus history from the Chiroptera to the Carnivora orders. J Virol 75: 8096-8104.

20. Sato G, Kobayashi Y, Motizuki N, Hirano S, Itou T, et al. (2009) A unique substitution at position 333 on the glycoprotein of rabies virus street strains isolated from non-hematophagous bats in Brazil. Virus Genes 38: 74-79.

21. Morimoto K, Patel M, Corisdeo S, Hooper DC, Fu ZF, et al. (1996). Characterization of a unique variant of bat rabies virus responsible for newly emerging human cases in North America. Proc Natl Acad Sci U S A 93: 5653-5658.
