# Supplementary material for: Molecular Inferences Suggest Multiple Host Shifts of Rabies Viruses from Bats to Mesocarnivores in Arizona during 2001–2009
Source: PLoS Pathog. 2012 Jun 21;8(6):e1002786. doi: 10.1371/journal.ppat.1002786 (PMC3380930; doi:10.1371/journal.ppat.1002786)
Supplement: Table S2 — Primers used in the present study. (DOC) [file ppat.1002786.s005.doc]

Table S2. Primers used in the present study.

| Primer name | Direction | Sequence | Position* |
| --- | --- | --- | --- |
| LYS001 | Forward | ACGCTTAACGAMAA | 1-14 |
| 304 | Reverse | TTGACGAAGATCTTGCTCAT | 1514-1533 |
| ENF | Forward | GATCACACATAAGGAGATATGTC | 1323-1345 |
| EMB | Reverse | CTATTCCAGAAGCATTGAAGAGTC | 3081-3104 |
| RVMF | Forward | ATGTCTCTTCAGACACAAAGGTC | 3045-3067 |
| 989 | Reverse | CTKAGACGTCTRAARCTYAC | 4259-4278 |
| G616 | Forward | ACHTCTTGTGAYATTT | 3932-3947 |
| 308d | Reverse | ACCTCTCCDGGATCGAKCAT | 5414-5433 |
| EGLF | Forward | CATCCCGATAAGGTGTGCTTAACT | 5294-5317 |
| L1918 | Reverse | AGBACAGARAATACATCCTC | 7307-7326 |
| L1790F | Forward | GATCGACAGAGTCACCGGACAGG | 7201-7223 |
| L4000 | Reverse | CCAGARACCATTCTRGATAT | 9443-9462 |
| L9320 | Forward | CGGCTGAAAGATTCTACCTTTCACTGG | 9326-9352 |
| L6220 | Reverse | GTAAATCARCCTKATCCAGTGAG | 11631-11653 |
| LYSEND | Reverse | ACGCTTAACAAAWAAA | 11913-11928 |

* The positions are related to SAD B-19 genome, GenBank accession No M31046.
